# Development of a sampling protocol for collecting leaf surface material for multiphase chemistry studies†

**DOI:** 10.1039/d4em00065j

**Published:** 2024-05-14

**Authors:** Rachele Ossola, Rose K. Rossell, Mj Riches, Cameron Osburn, Delphine Farmer

**Affiliations:** a Department of Chemistry, Colorado State University 80523 Fort Collins Colorado USA rachele.ossola@colostate.edu mj.riches@colostate.edu

## Abstract

Plant leaves and water drops residing on them interact with atmospheric oxidants, impacting the deposition and emission of trace gases and mediating leaf damage from air pollution. Characterizing the chemical composition and reactivity of the water-soluble material on leaf surfaces is thus essential for improving our understanding of atmosphere-biosphere interactions. However, the limited knowledge of sources and nature of these chemicals challenges sampling decisions. This work investigates how sampling variables and environmental factors impact the quantity and composition of water-soluble material sampled from wet leaves and proposes a flexible protocol for its collection. The ratio of solvent volume-to-leaf area, the solvent-to-leaf contact time, and environmental parameters – including the occurrence of rain, plant location and its metabolism – drive solute concentration in leaf soaks. Despite minor variations, UV-vis absorption spectra of leaf soaks are comparable to authentic raindrops collected from the same tree and share features with microbial dissolved organic matter – including overall low aromaticity, low chromophore content, and low average molecular weight. In addition to guiding the development of a sampling protocol, our data corroborate recent hypotheses on the amount, origin, nature, and reactivity of water-soluble organics on wet leaves, providing new directions of research into this highly interdisciplinary topic.

## Abstract 2

We describe a robust and flexible protocol to collect water-soluble chemicals from leaf surfaces for atmosphere-biosphere interaction studies.

## Graphical abstract

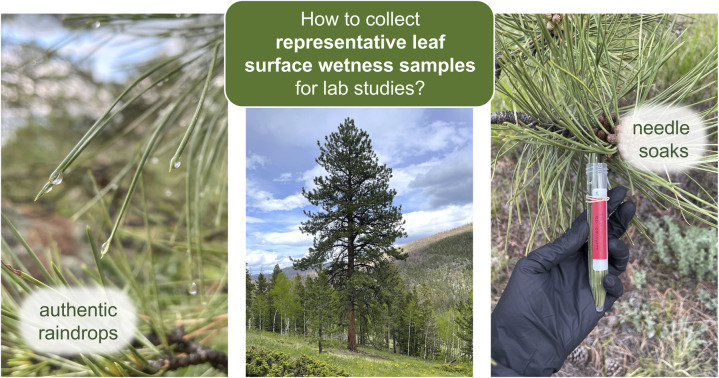

**Environmental significance**

Plant leaves occupy an area larger than the total land surface on Earth and are in constant contact with atmospheric oxidants. Although abundant evidence indicates that chemical reactions occur on these surfaces (especially when wet), the limited availability of empirical data on leaf surface chemicals hinders our understanding of these processes. In part, this knowledge gap stems from the absence of clear guidelines for collecting this material in an environmentally relevant manner. In this work, we develop a robust protocol to collect water-soluble chemicals from wet leaves that can be adapted to varying experimental needs and plant species. We hope these guidelines will favor comparability among studies and contribute to advancing this rapidly evolving field.

## Introduction

Plant leaves occupy an area larger than the total land surface on Earth^1^ and are in permanent contact with atmospheric oxidants – yet, for the most part, the atmospheric chemistry community has overlooked their role as multiphase reaction sites. Historically, leaf surfaces have only been considered in the bi-directional exchange of water-soluble gases such as sulfur dioxide and ammonia.^2–5^ This process involves gas uptake by leaf surface wetness and is controlled by pH and the co-occurrence of inorganic species.^2,3,6^ However, recent evidence has suggested that organic compounds also mediate the exchange of gas-phase chemicals.

Ozone (O_3_) is a striking example of atmospheric gases reacting with leaf surface organics. A recent review highlighted that more than 50% of observed dry ozone deposition on lands may be caused by non-stomatal uptake^7^ – that is, uptake through leaf surfaces other than stomata, pores that mediate plant-gas exchange. Ozonation of organic chemicals onto or within the leaf cuticle, the outermost layer of the leaf, is one of the most convincing explanations for this phenomenon.^7^ Two recent studies observed particularly high contributions of non-stomatal O_3_ uptake in plants with capitate glandular trichomes,^8,9^ and attributed it to ozonation of low-volatility organics produced by these structures and excreted onto the leaf. However, non-stomatal O_3_ uptake has also been described in *Acer rubrum*^12^ and *Quercus ilex*,^13^ plant species without glandular trichomes.^14,15^ In this case, O_3_ uptake was observed only in the presence of leaf wetness^12,13^ and, in *Q. ilex*, was explicitly associated with organic compounds.^13^ Beyond O_3_, surface reactivity may also help explain the bi-directional exchange of organic acids,^16–20^ nitrous acid,^21^ and peroxyacetyl nitrate^22–24^ from wet leaves.

The literature provides ample evidence for the presence of organic compounds that may participate in leaf surface reactions.^25^ These chemicals can be produced by the plant (endogenous) or deposited from the surrounding environment (exogenous). Examples of endogenous compounds include metabolites produced by glandular trichomes,^10,11^ resins,^26^ guttation fluids,^27^ and phyllosphere biofilms.^1,28,29^ Exogenous compounds include particulate matter,^30,31^ persistent organic pollutants,^32,33^ and chemicals delivered onto leaves through precipitation.^34^ A recent synthesis of the literature suggests that phyllosphere biofilms and particulate matter contribute 2–200 μg cm^−2^ of organic material on plant leaves, while other classes are less abundant or highly plant- and/or environment-specific.^25^ In addition, wet leaves may leach low-molecular-weight chemicals through cuticular water pores,^35,36^ a yet unrecognized (and thus unquantified) supply of organic matter on leaves.^25^ Our current knowledge of the amount and composition of leaf surface organics relies primarily on literature and indirect empirical data,^25,37^ highlighting the need for experimental evidence supporting their identity, concentration, and involvement in atmospherically relevant processes.

The first step in characterizing potentially reactive material on leaves is developing a protocol for its collection. The literature shows a rich variety of approaches that include, among others, infusing detached plant leaves into water,^38,39^ collecting water droplets or films from living leaves,^12,37^ or washing leaf blades with a small volume of water.^13^ Although these protocols are conceptually similar, sampling details vary considerably, with unforeseen impacts on chemical composition and environmental relevance. For instance, some authors created artificial wetness by spraying water or depositing water drops onto leaves,^12^ some obtained “leaf washes” by simply running water onto leaf blades,^13,40–42^ and others collected natural dew or rain drops using various tools (*e.g.*, metal spatulas,^37^ small vacuum pumps,^12,40^ syringes^43^) or approaches (*e.g.*, by dripping the liquid into a container^40^). Some authors immersed entire leaves,^39,44,45^ while others carefully kept the petiole out of the water.^38,46^ Additional considerations include the volume of liquid in contact with the leaves (*e.g.*, 5 to 500 mL),^44,46,47^ the solvent-to-leaf contact time (*e.g.*, “rinse” to 6 h),^13,39,45,46^ and the ionic strength of the sampling solvent (*e.g.*, deionized water *vs.* brine).^13,44^ Within this list, potentially relevant but unconstrained variables include the timescale of leaf-to-wetness interactions, the chemical composition of the solvent, the use of different collection equipment and strategies, and variations in leaf handling. Although we do not expect all variables to be relevant for every research question, ignoring their potential impact may cause one to collect and use material that is not environmentally relevant. Furthermore, this lack of knowledge makes it challenging to synthesize results from multiple studies.

To fill this gap, we tested how methodological choices impact the chemistry of material collected from wet leaves and developed a robust, repeatable, and flexible sampling protocol for its collection. This protocol mimics the conditions associated with non-stomatal O_3_ uptake at high relative humidity and the bi-directional exchange of water-soluble gases but minimizes confounding factors caused by variations in natural wetness and does not rely on precipitation. We first provide a general workflow for collecting and storing samples, including guidelines for selecting the most appropriate equipment and ensuring it is free of contaminants. We then evaluate the role of several sampling and environmental variables on the chemistry of the resulting sample. By analyzing authentic raindrops, we confirm that our protocol yields environmentally relevant material appropriate for leaf surface reactivity studies. Although we focus on a single ponderosa pine (*Pinus ponderosa*) to limit the number of uncontrolled variables, our findings agree well with the literature, making us confident of the general applicability of our guidelines. In the final section, we discuss how some of our data corroborate recent predictions on the quantity, nature, origin, and reactivity of leaf surface organics, highlighting potential directions of future research.

## Materials and methods

### Materials

Falcon tubes were purchased from Fischer Scientific (15 mL, 339650), Nalgene bottles from Thermo Scientific (60 mL, 16062-040), and syringe filters from Tisch Environmental (SF18238 and SF18249). All falcon tubes for sample collection were “pre-leached” by sitting filled with MilliQ water for ≥3 days at room temperature. The tubes were then thoroughly rinsed with new MilliQ water, air-dried, capped, and stored until use. Nalgene bottles were soaked for 3 hours in 1.2 M HCl, thoroughly rinsed with MilliQ water, air-dried, capped, and stored until use. MilliQ water used for tests, material cleaning, and dilution preparation for analyses was obtained from a Synergy Water Purification System (Millipore Sigma).

### Field sites

**Fig. 1:**
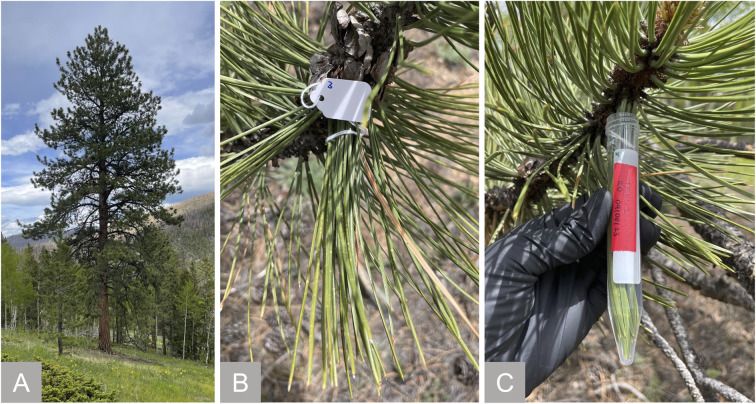
Ponderosa pine specimen from sampling at the CSU Mountain Campus on June 19, 2023 (A). In a typical soak, healthy needles growing in proximity were bundled together (B) and soaked for 5 min in 10 mL of MilliQ water in a pre-leached falcon tube (C).

We obtained most needle soaks and all raindrops from the lowest branches (≤3 m above ground level) of a single, mature ponderosa pine located at the North-East corner of the Colorado State University (CSU) Arboretum (Table S1†). The weather during the sampling period (May 22 to June 27, 2023) was wet until June 17 (172 mm of rain) and summer-like from June 18 onward (Text S1 and Fig. S1†). To evaluate plant-to-plant and spatial variability, we collected additional needle soaks from ponderosa pines in the CSU Mountain Campus (Fig. 1A), the Horsetooth Mountain Open Space, and the CSU Main Campus (outside the Arboretum). To limit day-to-day variability, these samples were obtained within 24 hours between June 18 and June 19. GPS coordinates, estimated tree age, and needle characteristics of all sampled specimens are in Table S1.†

### Sample collection and handling

A typical needle soak was prepared by first selecting 20 healthy needles in close proximity and bundling them together with cotton string (Fig. 1B). The bundle was then soaked for 5 min in a pre-leached falcon tube containing 10 mL of MilliQ water (Fig. 1C). Variations of this protocol allowed us to investigate the effect of specific sampling variables – *e.g.*, we modified the number of needles from 5 to 20, the soaking time from 5 seconds to 1 hour, and used natural rain instead of MilliQ water as the solvent (details in ESI†). In addition to soaks, we collected authentic raindrops during three rain events (Text S2 and Table S2†). In all cases, we handled needles with gloves to minimize contamination.

For each test, we collected one to five replicate samples that were either analyzed immediately (*i.e.*, within 4 hours) or frozen until analysis (maximum 2–3 weeks). For each sample, we measured total organic carbon (TOC), UV-vis absorbance, conductivity, and pH; samples for TOC analyses were diluted to match the instrument's volume requirements (details below). Before removing aliquots, falcon tubes sat vertical for at least 10 min to allow large particles to sediment (not analyzed). Although recommended in our final guidelines, samples were not filtered because we found negligible differences in bulk chemistry in filtered *vs.* unfiltered soaks (details below) and needed to optimize for a large number of samples rather than storage conditions.

Experimental blanks were obtained by filling pre-leached falcon tubes with MilliQ water; these tubes were prepared at the same time as the experimental soaks and treated as authentic samples. In selected tests, control soaks were prepared to evaluate specific hypotheses (details in ESI†).

### Analytical methods

#### Conductivity

Conductivity was measured with a compact conductivity meter (LAQUAtwin-EC-22, Horiba Scientific). Before measurements, we conditioned the probe for at least 1 h with the manufacturer's solution before calibrating with 1.41 and 12.1 mS cm^−1^ standards. We then washed the sensor with MilliQ water, dried it gently with a Kimwipe, and rinsed it with 120 μL of analyte. An additional 120 μL of sample was placed onto the sensor, the lid was closed, and the sensor was allowed to stabilize before recording the measurement. The sensor was generally unresponsive (*i.e.*, giving readings of 0 or 1 μS cm^−1^) below 5 μS cm^−1^, which we set as our “background” conductivity. Every 5–10 samples, we measured a 0.5 mmol L^−1^ potassium chloride solution to estimate precision (0–2.4%) and measurement error (*σ*_KCl_ ≈ ±0.8 μS cm^−1^). All solutions were allowed to equilibrate to room temperature before analysis.

#### UV-vis absorbance

Absorption spectra of needle soaks were acquired with a Spark Tecan multimode microplate reader equipped with a UV transparent 96-well plate (UV-Star 96-well microplate, VWR, 82050-778). Each run included at least three MilliQ water blanks, one reference sample, and the analytes (each 200 μL, filled with a calibrated pipettor; Fig. S2†). The plate reader was programmed to read spectra in triplicate between 200–800 nm at 1 nm steps using GRE 96-ft plates. All solutions were equilibrated to room temperature before analysis.

Raw data was processed in Matlab as detailed in Text S3 and Fig. S2–S3.† Briefly, for each run, we first obtained a mean blank spectrum (
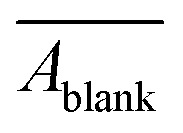
) by averaging all blank replicates. We then calculated the average of measurement triplicates, subtracted 
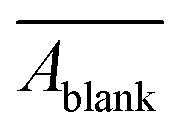
 from the resulting value, performed baseline correction, and divided by pathlength (*ℓ*) to obtain the decadic absorption coefficient (*α*, in cm^−1^). *ℓ* was determined from the reference sample's absorbance measured with the plate reader *vs.* a benchtop spectrophotometer equipped with a 1 cm pathlength quartz cuvette.

Each spectrum was further processed to yield metrics related to organic carbon quantity and quality – namely, the integrated absorption coefficient between 200 and 400 nm (∑*α*_200–400_, in cm^−1^), two absorption coefficient ratios (*α*_215_/*α*_200_ and *α*_260_/*α*_200_), the specific ultraviolet absorbance at 254 nm (SUVA_254_, in L mg_C_^−1^ m^−1^), and the spectral slope between 275 and 295 nm (*S*_275–295_). Details on these calculations and meaning of these parameters are in Text S3.†

#### Total organic carbon

TOC (non-purgeable total organic carbon) was quantified with a Shimadzu TOC-L analyzer (Shimadzu Scientific Instruments, Inc.). We injected 100 μL sample aliquots in duplicate or triplicate with sparge flow of 80, sparge time of 1 : 30 min, and 1.5% HCl addition. At the beginning of each run, we measured multiple calibration solutions (0.1–10 mg_C_ L^−1^), while every 5–10 samples we injected at least one MilliQ water blank and a control solution (exactly 2 mg_C_ L^−1^ in 2% HCl). We used the standard deviation of the blanks (*σ*_blank_) to calculate limits of detection (LoD = 3*σ*_blank_) and quantification (LoQ = 10*σ*_blank_), yielding LoD = 0.08–0.18 mg_C_ L^−1^ and LoQ = 0.25–0.59 mg_C_ L^−1^ (ranges are derived from multiple runs). Accuracy and precision were assessed *via* replicate measurements of the control solution, with respective values of 1.9% and 2.7%.

As the instrument required ≥20 mL of liquid, all samples were diluted gravimetrically in 40 mL acid-cleaned glass vials using new pipettor tips, acid-cleaned glassware, and an analytical balance (±0.1 mg). Controls confirmed that our dilution protocol neither introduced contamination, nor increased the measurement error.

#### Other methods

In samples above background conductivity, we quantified the solution pH with a portable probe. In the test investigating material leaching from stomata, we quantified stomatal conductance, a metric of stomata openness,^48^ with a portable photosynthesis system (LI-COR 6800). Due to volume limitations, absorption spectra of authentic raindrops were measured using a Tecan NanoQuant plate rather than a 96-well plate. Details on these methods are in Text S4 and Fig. S4.†

### Data treatment

Averages and standard deviations are from three to five sample replicates collected over the same or multiple days. These values were used as inputs for unpaired *t*-tests,^49^ which helped us identify statistically significant variations between treatments (*p* < 0.05). When samples were collected over multiple days, we also evaluated normalized averages to uncover potential trends overshaded by day-to-day variability. Normalized averages were obtained by first computing *y*^*R*_i_^/*y*_ref_^*R*_i_^ for each replicate (*y*_ref_^*R*_i_^ is the reference value of a generic variable *y* in the replicate *R*_i_) and then calculating average and standard deviation of the three or five *y*^*R*_i_^/*y*_ref_^*R*_i_^ values. The reference sample varied from test to test as detailed in the ESI.† In this case, changes are considered substantial when the average *y*^*R*_i_^/*y*_ref_^*R*_i_^ value ± its standard deviation is different from 1. In the “solvent volume-to-leaf area ratio” and “solvent-to-leaf contact time” tests, parameters are considered constant when they show no consistent trends as a function of needle number or soaking time, respectively (*i.e.*, *R*^2^ < 0.5 and/or value within the average range for needle soaks; details in ESI†).

## Results

### General workflow for sample collection and storage

#### Sample type selection

This work aims to develop a flexible protocol to collect material naturally present in leaf wetness for atmosphere-biosphere interaction studies. We focus on water-soluble organics due to their anticipated role in mediating the dry deposition of ozone and other trace gases onto wet plant leaves. To this end, we collected “soak” samples – *i.e.*, we immersed living leaves into a fixed volume of MilliQ water for a few minutes (Fig. 1). This approach is analogous to the standard way to collect leaf-adsorbed particulate matter (*e.g.*, Ristorini *et al.*^39^) but differs in that we minimize plant damage by not detaching the leaves. Furthermore, it mimics the “long” wetness residence time observed under natural settings without requiring the occurrence of precipitation – thereby overcoming the limitations of other two approaches, namely “washing” living leaves with water^13^ (too short contact time relative to natural conditions, which involve water interacting with surfaces for minutes to hours^50^) and collecting dew or raindrops^12,37^ (relies on meteorological conditions and requires an understanding of rain/dew chemistry). As we clarify in the discussion, our protocol additionally allows one to increase sample volume and/or concentration to meet experimental needs, which is hard to achieve with the latter two methods.

#### Workflow for soaks collection and storage

**Fig. 2:**
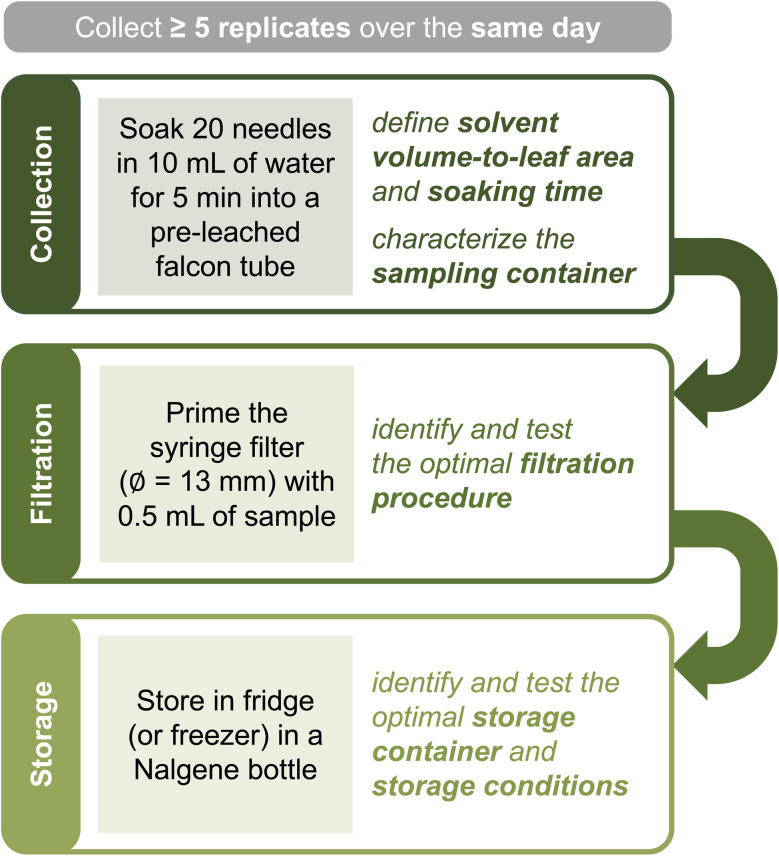
Proposed workflow for sample collection and storage. The text in the shaded squares outlines typical conditions for collecting ponderosa pine needle soaks. Filtration can be skipped if sample volume is insufficient or experiments require unfiltered material. An ideal sampling strategy involves collecting ≥5 replicates over the same day.

Fig. 2 proposes a workflow for the collection and storage of soak samples that maximizes sample lifetime while minimizing potential contamination from containers or sample handling. We recommend using different containers for collection and storage – the former chosen to optimize the ratio of solvent volume-to-leaf area, the latter selected to limit contamination from the vial itself when samples are left for long periods of time. We also recommend filtering samples right after collection to improve preservation and limit measurement artifacts. All material used throughout the process should be tested for potential interferences with any analyses beyond those described herein.

In this work, we focused on ponderosa pines, common conifers of the Colorado Front Range characterized by long (≈10–20 cm) and thin (1.2–2.2 mm in diameter) needles (Fig. 1 and Table S1†). Because of the unique leaf morphology, we found 15 mL falcon tubes were ideal sampling containers for this species (Fig. 1B–C). In general, we soaked 5–20 living needles in 10 mL of solvent, equivalent to ≈1.3–5.3 cm^2^ mL^−1^. As we show below, the resulting samples are in appropriate concentration ranges for our chosen analytical methods. The rationale for selecting needle number, solvent type, soaking time, number of replicates, and sampling conditions is discussed below.

While conveniently shaped for ponderosa pine needles, brand new falcon tubes leached a measurable amount of organic carbon when in contact with water ([TOC] = 0.7 mg_C_ L^−1^ and ∑*α*_200–400_ = (6.2 ± 0.8) cm^−1^ after 14 days of being filled with MilliQ water at room temperature; Fig. S5A†). Although this quantity is small compared to a typical needle soak (Table S3†), “pre-leaching” the tubes – *i.e.*, letting them sit for >3 days filled with MilliQ water – reduced this contamination considerably ([TOC] < LoQ and ∑*α*_200–400_ = (1.1 ± 0.9) cm^−1^ for MilliQ water placed in tubes for 1 month after an initial pre-leaching; Text S5†). For this reason, we collected needle soak samples solely in pre-leached containers.

As soon as possible after collection, we recommend filtering the sample into a storage container with 0.2 μm pore filters to remove most microorganisms and thus preserve the chemical composition of the organic matter present.^51,52^ If filtration is not viable and the sampling vessel is not entirely free of contaminants, we still advise transferring the sample into a storage vial. We found syringe filtration with 1.3 mm diameter filters to be ideal for low sample volumes (≤10 mL), as this filter size requires ≤0.5 mL for priming (Text S5†). If relevant, the >0.2 μm fraction can be recollected from filters (*e.g.*, through backflushing^53^) and stored for further use. The filtrate should be collected into a contaminant-free container for storage. High-density polyethylene (Nalgene) or polycarbonate bottles are ideal for both organic and inorganic components; if focusing on organics, pre-combusted glass containers are an excellent alternative.^54^ Storage containers should be tested for both leaching and wall adsorption before use. According to our tests, storing the filtered and unfiltered soaks in the fridge or freezer (for over two months and five freeze-thawing cycles) works equally well in preserving bulk sample properties (Text S5†).

### Bulk chemistry of needle soaks and comparison with authentic raindrops

Before discussing the details of the sampling protocol, we give an overview of the chemistry of ponderosa pine needle soaks to clarify their environmental relevance and provide context for discussion. For simplicity, the average data presented below is based on 20-needle soaks from the specimen in the Arboretum – though these trends were consistently observed throughout the dataset (Text S6 and Fig. S6†).

We found a remarkable similarity in bulk chemical properties among samples. Both organic and inorganic water-soluble species were always present in concentrations of 1.6–74 mg_C_ L^−1^ and 1–27 μS cm^−1^, respectively (Table S3†), with sporadic outliers as high as 150 mg_C_ L^−1^ (Fig. S6A†) and 70 μS cm^−1^. Absorption spectra were always distinct from the blanks, with ∑*α*_200–400_ = 3.2–38 cm^−1^ (Table S3†). We observed a significant positive correlation between conductivity and ∑*α*_200–400_ (*R*^2^ = 0.84; Fig. S6B†), while the trend was less strong for TOC – primarily due to a few samples with exceptionally high organic carbon content (Fig. S6A†). All samples were slightly acidic (pH of 5.7 ± 0.5) and contained nitrogen, primarily in its organic form (≈70%; Table S3 and Text S6†). Both total and organic nitrogen correlated strongly with conductivity and ∑*α*_200–400_ (*R*^2^ > 0.74; Fig. S6C and D†). In general, we did not detect particulate matter, although we observed insoluble residues in some samples. These residues sedimented within minutes when tubes were left vertical and were never included in further analyses. Although overall more concentrated (*e.g.*, [TOC] ≈ 10 to >900 mg_C_ L^−1^; Table S2†), authentic raindrops collected from the needles of the same ponderosa pine also contained organic carbon, nitrogen, and inorganic species and their pH was comparable to needle soaks (Text S2 and Table S2†).

**Fig. 3:**
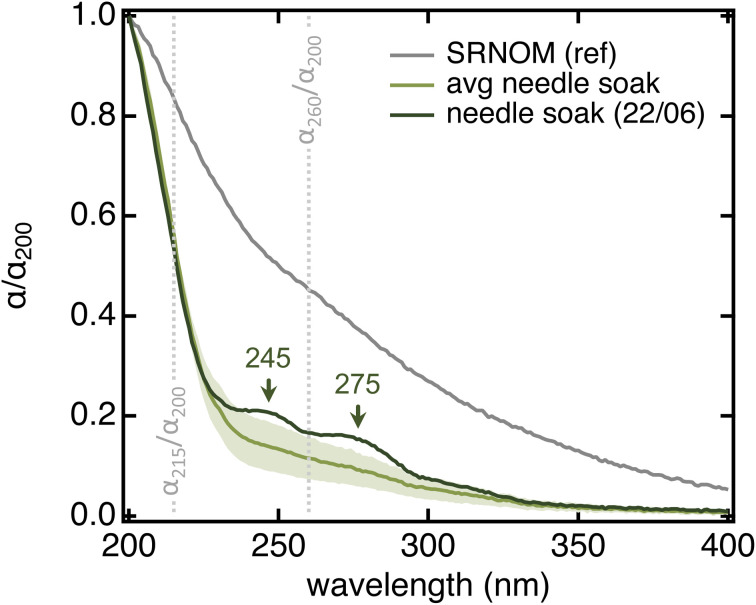
Absorption spectra (normalized by their absorption at 200 nm) highlighting differences between SRNOM (grey) and ponderosa pine needle soaks (green). The grey trace is the average of all SRNOM samples used as reference in 96-well plate measurements (Fig. S3A,†*N* = 12). The light green trace is the average of all 20-needle, 5 min soaks in MilliQ water that we collected from the Arboretum specimen during this project (*N* = 28; details in Text S6†); shading represents the standard deviation. Qualitative organic matter parameters associated with these two spectra are in Table 1. The dark green trace is one of the two soaks (out of the 28) where the discrete absorption features were evident. The grey vertical lines identify the two absorption ratio parameters that we describe in the text.

All needle soaks were remarkably similar in terms of spectral properties and overall comparable to authentic raindrops collected from the same tree while being statistically different from Suwannee River Natural Organic Matter (SRNOM), a dissolved organic matter (DOM) sample from terrestrial aquatic environments (Table 1 and Fig. 3). In particular, needle soaks and raindrops collected from the same ponderosa pine had lower aromaticity (*i.e.*, lower SUVA_254_) and lower average molecular weight (*i.e.*, higher *S*_275–295_) than SRNOM (*p* < 0.0004 for all parameters) – in fact, they were comparable to microbial and/or open-ocean DOM (SUVA_254_ < 2 L mg_C_^−1^ m^−1^, *S*_275–295_ > 0.020 nm^−1^).^55–57^ Authentic raindrops appeared slightly more aromatic than needle soaks, potentially because of the lag between raindrop deposition and collection (>3 hours; details in Text S2†). We note that also DOM in throughfall (*i.e.*, bulk rain collected below tree crowns; also referred to as tree-DOM) is more aromatic (SUVA_254_ = 1.6–3.3 L mg_C_^−1^ m^−1^) and has a lower spectral slope (*S*_275–295_ = 0.014–0.017 nm^−1^) than needle soaks, features that have been associated with chemical processes occurring between deposition to the biosphere and sample collection.^58,59^ In <10% of the soaks, we noticed discrete absorption features at 245 and 275 nm (Fig. 3) that are consistent with a significant abundance of one (or a few) individual compound(s). A peak at 275 nm was also observed in the composite raindrop sample with the highest *α*_260_/*α*_200_ and SUVA_254_ values (Table S2†). We suspect these features correspond to needle damage (see below). In all other cases, the absorption spectrum showed a smooth bi-exponential decay.

**Table 1:** Qualitative organic matter parameters for soaks prepared with living ponderosa pine needles, authentic raindrops collected from the same tree, and Suwannee River natural organic matter (NOM). Data for soaks are averages of all samples collected from the specimen in the Arboretum using 20 needles, 5 min soak time, and MilliQ water as solvent (details in Text S6). Absorption spectra parameters for raindrops and SRNOM are obtained as outlined in Text S2 and Text S3, respectively

	Needle soaks	Raindrops collected from needles	Suwannee river NOM^e^
*α*_215_/*α*_200_	0.56 ± 0.02	0.58 ± 0.04	0.84 ± 0.04
*α*_260_/*α*_200_	0.12 ± 0.04	0.17 ± 0.03^c^	0.46 ± 0.06
SUVA_254_ (L mg_C_^−1^ m^−1^)	1.0 ± 0.5	1.40 ± 0.06^a^	4.2^b^
*S*_275–295_ (nm^−1^)	0.024 ± 0.006	0.018 ± 0.003^d^	0.014 ± 0.001
*N*	28	8	12

a Average of 3, not 8 values (see Table S2).

b TOC was quantified only once, thus the error is not reported. For the *t*-test, we assume an error of 0.5 L mg_C_^−1^ m^−1^.

c Statistically different from soaks (*p* = 0.0025).

d Statistically different from soaks (*p* = 0.010).

e All parameters statistically different from soaks and raindrops (*p* < 0.0004).

### Sampling variables

A central objective of this work is to identify and subsequently optimize the sampling parameters that will yield an accurate mimic of natural leaf wetness in terms of bulk composition. Taking inspiration from the literature, we compiled a list that includes variables related to the solvent, the soaking approach, and features of the leaf (Table 2). In general, we found only the ratio of solvent volume-to-leaf area and the solvent-to-leaf contact time to significantly influence the amount of organic and inorganic species in needle soaks; leaf damage and treatment like sonication may additionally impact needle soak's chemistry both qualitatively and quantitatively. All other tested variables had negligible or no effects. Below, we describe in detail the most significant findings; a full report of each test is in Text S7.†

**Table 2:** Investigated sampling variables and their effect on soak needle chemistry (dissolved species only). The criteria used to assess whether a variation is significant or not are outlined in the Materials and methods; variables in parenthesis were found to change substantially only after correcting for day-to-day variability (see Text S7 for details)

	Variations in …		Reference data
	Quantity	Quality	
**Solvent**			
Solvent volume-to-leaf area ratio^b^	TOC, ∑*α*_200–400_, cond	No^e^	Fig. S7
Rain *vs.* MilliQ water	No^f^	No	Table S4

**Sampling approach**			
Solvent-to-leaf contact time^a^	TOC, ∑*α*_200–400_, cond	(*α*_260_/*α*_200_, SUVA_254_)	Fig. 4, S8 and S9
Sonication^a^^c^	(∑*α*_200–400_)	*α*_260_/*α*_200_, (SUVA_254_)	Fig. S10 and Table S5
Filtration^b^	No	No	Table S6
Freezing^a^	No	No	Table S7

**Leaf**			
Healthy *vs.* damaged^a^^d^	∑*α*_200–400_, cond, (TOC)	*α*_260_/*α*_200_	Table S8
Living *vs.* dead^a^	No	*S*_275–295_	Table S9
On-plant *vs.* detached^a^	No	No	Table S10
Open *vs.* close stomata	No	No	Table S11

a Sample replicates collected over different days.

b Using samples and/or data collected for other tests.

c Only results for living needles; data for dead needles are in Table S5.

d Living needles, detached.

e SUVA_254_ may decrease slightly with leaf area; see main text.

f The difference in conductivity is fully justified by solvent chemistry.

#### Solvent volume-to-leaf area ratio

The solvent volume-to-leaf area ratio impacted the mass of material extracted during the soak. Fig. S7A–C† shows the correlation between TOC, ∑*α*_200–400_, and conductivity as a function of needle number (*i.e.*, leaf area) for samples collected between June 6 and 8 in the context of other experiments (Text S7†). Despite large error bars, this correlation is excellent for the three parameters (*R*^2^ ≥ 0.994), showing that more organic and inorganic species were released in solution as leaf area increased. This linearity further indicates that soak data can be normalized to surface area, providing a pathway forward for comparing measurements across studies. The type of organic material did not vary with needle number, as evidenced by the lack of change in *α*_215_/*α*_200_, *α*_260_/*α*_200_, and *S*_275–285_ (*R*^2^ = 0.0004–0.086; Fig. S7D and F†). SUVA_254_ was the only qualitative parameter that varied significantly (Fig. S7E†) – but given the limited data, large error bars, and the fact that all values were within the average SUVA_254_ for ponderosa pine needles, differences in aromaticity were minor.

We attribute the large replicate uncertainty to varying environmental conditions. This fact is particularly evident when considering the three 20-needle soak replicates, two of which were collected on June 6 and one on June 8 – approximately 14 and 62 h, respectively, after the last rain event (Text S1†). Notably, all quantitative parameters were substantially lower for the June 6 as compared to the June 8 replicates (*i.e.*, −69% in TOC, −67% in ∑*α*_200–400_, and −27% in conductivity).

#### Solvent-to-leaf contact time

**Fig. 4:**
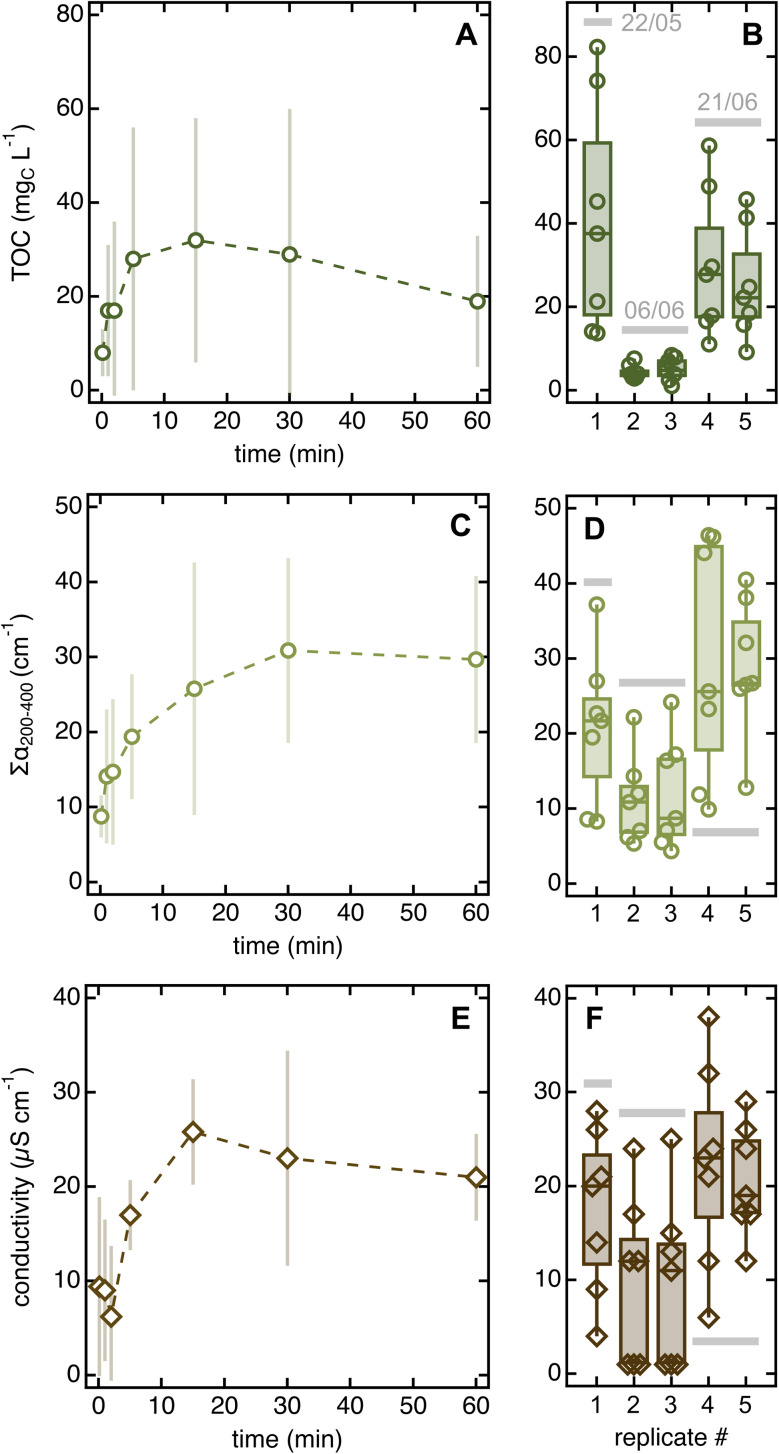
Changes in TOC (A), ∑*α*_200–400_ (C), and conductivity (E) as a function of solvent-to-leaf contact time. Datapoints are averages of five replicates collected on three different days (May 22, June 6, and June 26) and error bars are standard deviations. The boxplots on the right highlight the large day-to-day variability in all parameters (panels (B, D and F)). Fig. S8A–C† show the same data corrected for day-to-day variability. Qualitative organic matter parameters and pH are in Fig. S8D–F and S9.†

The amount of organic and inorganic material released during soaking first increased and then plateaued (or decreased slightly) as a function of time (Fig. 4 and S8A–C†), while organic matter quality and pH did not change considerably (Fig. S8D, E and S9†). The non-linear role of soaking time in impacting concentration carries implications for comparing data across studies.

Error bars for solvent-to-leaf contact data are large, which we interpreted as reflecting day-to-day variability rather than lack of significance in the experimental trends. Two observations justify this conclusion. First, the two replicates collected on June 6 (∼14 h after rain) had consistently lower integrated absorbance, conductivity, and TOC than those taken on other sampling days (>80 h after rain; Fig. 4B, D, and F). Second, when normalized by the daily 5 seconds data, all error bars decreased consistently but the trends remained the same (Fig. S8A–C†).

The existence of trends as a function of soaking time further suggests that distinct pools of chemicals are released on different timescales and that reactions may occur after leaching. Indeed, all 5 seconds soaks contained more material than MilliQ blanks, indicating that chemicals are released in solution within extremely short contact times. According to UV-vis absorption data, the loosely associated organic species leached in rapid soaks are slightly different from those released in prolonged dips, which appear slightly more aromatic (*i.e.*, they have slightly higher daily-normalized *α*_260_/*α*_200_ and SUVA_254_ values than 5 seconds soaks; Fig. S8D and E†). Once in solution, organics may undergo rapid reaction, as suggested by the decreasing TOC (Fig. 4A) but constant integrated absorbance (Fig. 4B) for soaking times >15 min. This result hints that aliphatic species absorbing <200 nm (*i.e.*, not visible in the absorption spectrum) degrade after prolonged soaks, which we speculate is due to the phyllosphere feeding on these compounds. This degradation process may be enhanced by water abundance and, potentially, temperature increases caused by hand-holding the sampling tubes. An alternative (yet untested) hypothesis is that aliphatic compounds get preferentially adsorbed onto the test tube walls during long dips.

#### Other sampling variables

Although the solvent volume-to-leaf area ratio and soaking time were the two most relevant sampling variables, leaf damage and sonication also influenced needle soak chemistry. To evaluate the impact of leaf damage, healthy needles were collected, their tips were broken off, and the bulk chemistry of the resulting soak was compared to a control prepared with unbroken needles. Soaks from damaged needles had significantly higher ∑*α*_200–400_ (*p* < 0.0001) and lower *α*_260_/*α*_200_ (*p* = 0.008) than healthy ones (Table S8†). After correcting for day-to-day variability, we found that also TOC was substantially higher in damaged needles (Table S8†), hinting that they release more, and potentially different, organics as compared to healthy ones. All five replicates obtained with damaged needles had an absorption feature at 275 nm that was never observed in the controls – although it was occasionally seen in soaks from the same tree (Fig. 3) and authentic raindrops (Table S2†).

We found that sonication increased dissolved organics at the expense of needle integrity (Text S7†). This effect was particularly evident for dead needles, for which we detected a significant increase in ∑*α*_200–400_ and *α*_215_/*α*_200_ (*p* = 0.014 and 0.038, respectively; Table S5†), the occasional presence of absorption features at 245 and 275 nm, and visual evidence of needle damage (Fig. S10†). Although less evident, changes in absorption spectra were also observed in sonicated living needles (Table S5†).

Using natural rain instead of MilliQ as solvent (Table S4†), employing living (detached) *vs.* dead needles (Table S9†) or on-plant *vs.* detached (living) needles (Table S10†), keeping stomata closed during sampling (Table S11†), and storing detached needles in the freezer before preparing a soak (Table S7†) had no or negligible effects on needle soak chemistry.

### Environmental variables

While investigating the role of sampling variables, we noticed a systematic trend in soaks collected from the same tree and location but on different days. This observation prompted us to analyze how environmental variables – *i.e.*, meteorological conditions, location, and plant-to-plant variability – impact soak chemistry.

#### Meteorological conditions

**Fig. 5:**
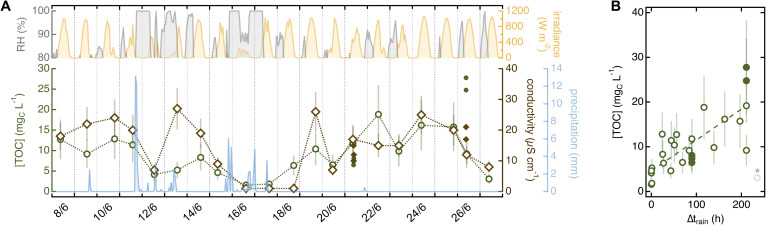
(A) Correlation between meteorological conditions and chemistry of soaks collected from the ponderosa pine in the Arboretum between June 8 and June 27 (20 needles in 10 mL of MilliQ water for 5 min). The top shows trends in relative humidity (RH; grey; only value >80%) and irradiance (yellow). The bottom presents variation in TOC (green) and conductivity (brown) overlaid to cumulative precipitation (light blue). ∑*α*_200–400_ follows the same trend as conductivity (*R*^2^ = 0.90) and is shown in Fig. S11.† Filled dots are 20-needle soaks collected on the same tree for different tests. Datapoints are individual sample replicates and error bars are estimated from average relative errors as outlined in Text S8.† Meteorological data was from the Fort Collins Weather Station located on campus (Text S1†).^61^ (B) Variation in TOC as a function of time after rain (>0.5 mm; Δ*t*_rain_). Data representation is the same as panel A; the dashed line is a regression using all values with Δ*t*_rain_ < 215 h (*R*^2^ = 0.56). The last datapoint (shaded and marked with an asterisk) is excluded from the analysis. Similar plots for other variables are in Fig. S13.†

Meteorological conditions impacted the amount – and potentially type – of organic and inorganic species in needle soaks. Daily samples from the Arboretum ponderosa pine showed defined trends with meteorology for TOC, ∑*α*_200–400_, and conductivity (Fig. 5A and S11†), while absorption spectra parameters and pH remained overall constant (Fig. S12†). Further data processing unraveled correlations with time after the last significant precipitation event (*i.e.*, >0.5 mm; Δ*t*_rain_) for almost all variables, at least for Δ*t*_rain_ ≤ 215 h (≈9 days). This correlation was most significant for TOC (*R*^2^ = 0.56; Fig. 5B), while for ∑*α*_200–400_ and conductivity, we only detected a tendency for minimum values to increase with Δ*t*_rain_ (Fig. S13A and B†). The mismatch between TOC and ∑*α*_200–400_ indicates this trend to be driven by aliphatic organics absorbing <200 nm. Additionally, most qualitative parameters consistently decreased with Δ*t*_rain_, including SUVA_254_ (*R*^2^ = 0.42), pH (*R*^2^ = 0.36), *α*_215_/*α*_200_ (*R*^2^ = 0.25), and *α*_260_/*α*_200_ (*R*^2^ = 0.24; Fig. S13C–E†). This decrease was always within the range of typical needle soak values (Table 1), strengthening the idea that meteorological conditions are important drivers of needle soak chemistry. Notably, rainfall was also found to influence the molecular composition of dew and frost samples collected from leaf blades^37^ and the leaching of nutrients from tree canopies.^60^

#### Location and plant-to-plant variability

**Fig. 6:**
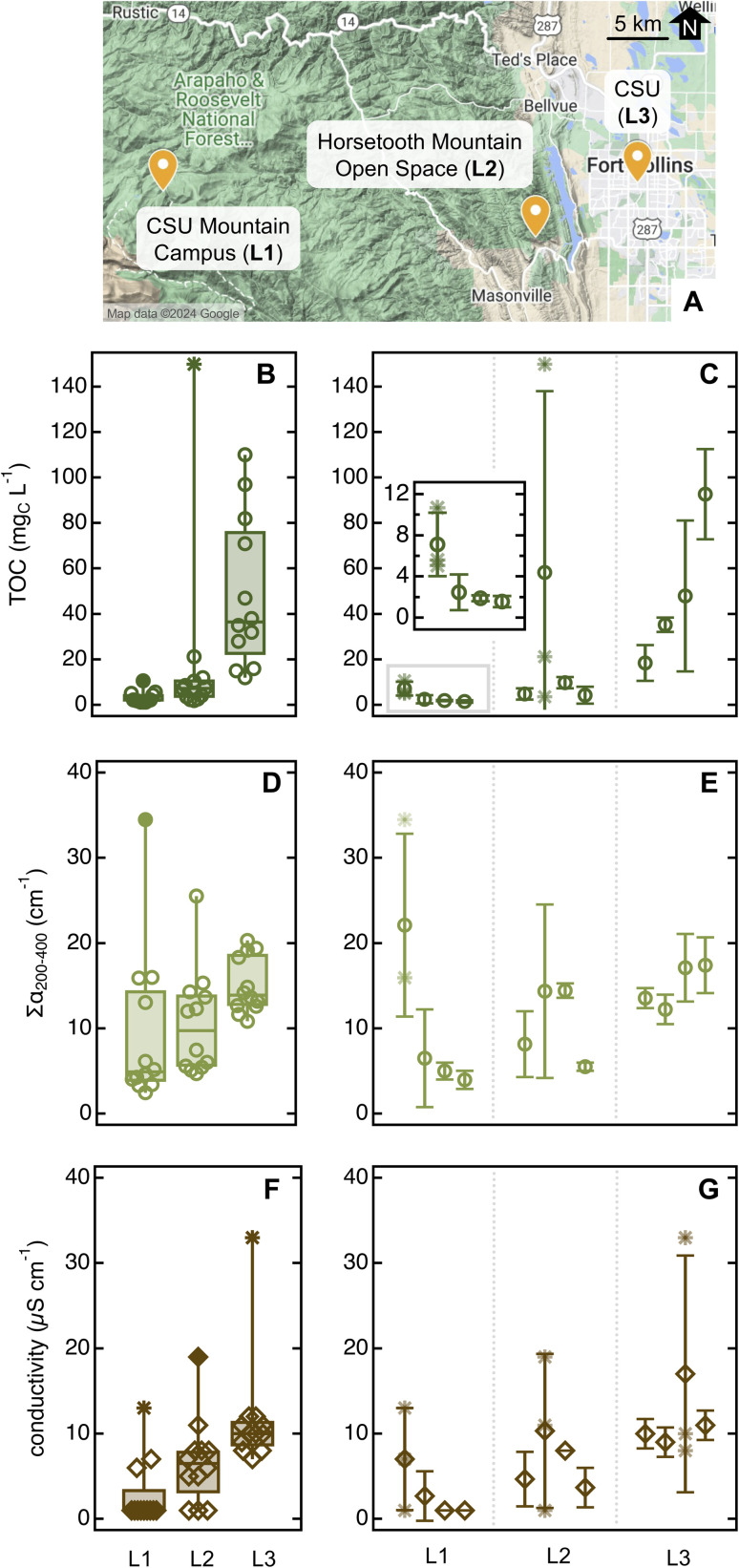
Overview of sampling locations ((A); map obtained from Google My Maps). Descriptive statistics for TOC (B), ∑*α*_200–400_ (D), and conductivity (F) for ponderosa pine needle soaks (20 needles in 10 mL of MilliQ water for 5 min). Filled data and asterisks are outliers and far outliers, respectively (data plotted with Igor 8). Averages for each parameter in each location are in Table S12.† Panels (C), (E), and (G) report average and standard deviation for TOC, ∑*α*_200–400_, and conductivity, respectively, for individual ponderosa pines; the insert in panel (C) is a zoomed view for trees in L1. Here, asterisks denote individual sample replicates in trees that presented at least one sample outlier (as identified by the descriptive statistic on the left panels). As a comparison, the ponderosa pine in the Arboretum had [TOC] = 8.4 mg_C_ L^−1^, ∑*α*_200–400_ = 21 cm^−1^, and conductivity = 14 μS cm^−1^ (data from Fig. 5 and S11,† average for June 18 and 19, 2023).

Lastly, we investigated how soak chemistry varies across locations and among individual plants. To this aim, we sampled various plants in the CSU Mountain Campus, Horsetooth Mountain Open Space, and CSU Main Campus in Fort Collins, which lie along a remote-to-urban gradient within ≈50 km radius (Fig. 6A). All samples were collected within 24 hours between June 18 and 19, which were dry weather days in all locations (Text S1†).

The concentration of organic and inorganic solutes increased significantly from pristine to urban sites (Fig. 6 and Table S12†). This enhancement was especially marked for TOC (Fig. 6B; *p* ≤ 0.0003), which we attributed to aliphatic organics absorbing <200 nm released only by individuals in urban areas – as ∑*α*_200–400_, also a proxy for organics, did not show a similarly conspicuous increase. The average SUVA_254_ was significantly lower in the unmanaged urban trees ((0.20 ± 0.17) L mg_C_^−1^ m^−1^) than specimens in other locations (>0.7 L mg_C_^−1^ m^−1^, *p* ≤ 0.0003; Table S12†) – including the reference tree in the Arboretum, which is urban but well managed ((1.0 ± 0.5) L mg_C_^−1^ m^−1^, *p* < 0.0001; Table 1). *S*_275–295_ was low in unmanaged urban specimens ((0.012 ± 0.004) nm^−1^*vs.* >0.020 nm^−1^; Tables 1 and S12†) although probably due to organics associated to needle damage, not due to an increase in average molecular weight. Indeed, 33% of the samples collected in this location showed a clear peak at 310 nm that impeded spectral slope calculation. While sampling at the unmanaged urban site, we also noted aphid-like insects (and/or their eggs) and had more difficulty in finding bundles of healthy-looking needles (*i.e.*, without brown tips and/or yellow spots) than in other sites, including the nearby Arboretum. Insect infestations affect plant physiology, emission of volatile biogenic organics,^62^ and organic carbon content in tree-DOM,^59,63^ and it is perhaps unsurprising that they impact leaf soak chemistry. Together, these observations lead to the hypothesis that the unmanaged urban trees were less healthy than those sampled at rural or managed urban sites. The difference between managed and unmanaged pines growing only tens of meters apart is particularly striking and suggests that plant health is more relevant than background pollution in driving needle soak properties.

Further supporting this last statement, individual trees showed variability within each location. For example, the first plant sampled in the remote site is an outlier for all three quantitative parameters (Fig. 6C), in addition to having *α*_215_/*α*_200_ and *α*_260_/*α*_200_ substantially higher than the reference pine and other plants at the same location (Fig. S14A†). While sampling, we noticed that this pine was sprouting new needles, which is known to impact leaf physiology^64,65^ and the leaching of nutrients from wet leaves.^60^ At the Horsetooth Mountain Open Space, we found the second plant to be an outlier for TOC and conductivity, while in the urban site, it was the third pine that was an outlier for conductivity (Fig. 6). Thus, both plant-to-plant and needle-to-needle variability is present, underscoring the importance of taking an adequate number of replicates for high-quality data.

## Discussion

### Guidelines for sampling leaf surface chemicals

Given the impact of the solvent volume-to-leaf area ratio and solvent contact time on soak chemistry, using a consistent sampling approach is essential for creating intercomparable datasets. We recommend following the general workflow in Fig. 2 if interested in collecting water-soluble material as the one found in authentic leaf wetness. As collection parameters, soaking 20 needles (≈50 cm^2^) in 10 mL of solvent for 5 min in a falcon tube was an ideal combination for our selection of plant species, analytical methods, and experimental needs. This recipe may need adjustments to meet different research goals. If interested in more concentrated soaks, one can increase the leaf area while keeping the solvent volume constant or perform “sequential soaking” – *i.e.*, soak several groups of 20 needles into the same tube. The soaking time can be varied to access different pools of chemicals – though organics may undergo reactions after 15–20 min of soaking (under our sampling conditions; Fig. 4). Furthermore, while falcon tubes may be appropriate for other plants with long leaves (*e.g.*, some *Eucalyptus* or grass species), other morphologies may require a different container that optimizes the solvent volume-to-leaf area ratio. The sampling vessel should be tested for suitability, contamination, and wall loss beforehand. In general, solvent volume-to-leaf area ratio and soaking time are two parameters that must be specified to assure replicability.

In addition to sampling variables, environmental conditions affect soak chemistry. We recommend collecting ≥5 sample replicates to confidently parse apart minor differences in chemical properties – fewer replicates are generally insufficient due to high prevalence of outliers. One should be mindful of meteorological conditions, especially occurrence of rain and/or other factors that may induce natural formation of leaf wetness, and season.^59^ Soak chemistry changes among plants growing nearby (Fig. 6) – thus, depending on the specific research question, one may want to collect replicates from the same plant or from different individuals, within one day or across several days, within a short time span or throughout the year.

Specific variants of the protocol in Fig. 2 will yield soaks with the same bulk chemistry as described in this work. Notably, samples prepared in the lab with detached needles (right after collection or after storage for up to 2 days at −20 °C) were undistinguishable from those obtained with living needles in the field (Tables S7 and S10†). Thus, if plant damage is not an issue, one can collect leaves and manipulate them in the lab, where soaking conditions (*e.g.*, solvent temperature) are more reproducible and leaf area can be more accurately assessed.

While we are confident of our findings, our work has two important caveats. First, our conclusions are based on bulk analyses and may not be directly translatable to individual species. For example, as some ions are more efficiently released than others from wet leaves (*e.g.*, Mn^2+^*vs.* Na^+^),^66,67^ the use of rain as soaking solvent (instead of MilliQ water) may be advisable in specific cases.^60^ Thus, research questions that focus on selected organic or inorganic species may require adjustments to the general protocol. Second, our conclusions are based on samples collected from a single plant species. While the overall behavior of ponderosa pine needle soaks fits well with the literature (see below), specific details (*e.g.*, soaking time and sampling container) may require prior testing and/or adjustments when investigating different species.

### Insights into the origin and reactivity of chemicals in leaf wetness

Although not our primary research objective, the experiments described in this work provided valuable insights into the nature and reactivity of leaf-surface chemicals. Below, we summarize our main results and provide directions for future studies.

#### A substantial amount of chemicals is available for leaf surface reactions

We unambiguously showed that dissolved species are released from leaves in contact with water. For organics, we estimated this amount to be 0.6–28 μg cm^−2^, with peaks as high as 57 μg cm^−2^ (Text S6†) – numbers within literature ranges for total and non-particulate organics on leaf surfaces (2–200 and 1–90 μg cm^−2^, respectively).^25^ Particles >2.5 μm are posited to dominate the mass of non-soluble organics on leaves;^25^ consistently, we observed insoluble deposits in our sampling tubes but failed to detect suspended particles bigger than 0.2 μm (Table S6†). Although unquantified, these “large” particles are likely to bring sizeable contributions to the total leaf surface mass of organics, potentially making the 2–200 μg cm^−2^ range an underestimate.

#### Dissolved chemicals in needle soaks are primarily endogenous

Several pieces of evidence indicate that dissolved chemicals in ponderosa pine needle soaks come from phyllosphere biofilms, cuticular water pores, or both – thus, as recently hypothesized,^25^ they are primarily endogenous (mature conifers do not have glandular trichomes^68^ and we expect guttation to be negligible under our sampling conditions;^69^ these contributions are thus neglected in the discussion below). First, the amount of soluble organics in needle soaks matches well with estimated inputs from extracellular polymeric substances (EPS; 1–90 μg cm^−2^),^25^ major non-particle components of phyllosphere biofilms.^70,71^ Second, our trends in quantitative chemistry parameters as a function of soaking time (Fig. 4) agrees surprising well with the kinetics of water absorption from isolated cuticles,^72,73^ the first step to create cuticular water channels.^35,74^ Due to the polymeric structure, leaching of individual chemicals from EPS may also require a lag time, *e.g.*, to allow monomers to hydrolyze or small molecules to diffuse out of the polymer. Third, absorption spectra parameters reliably indicate needle soaks to contain “microbial-like” organics of low average molecular weight (Table 1). The presence of “microbial-like” features strength the phyllosphere hypothesis, while low average molecular weight is a requisite for material leached through water pores^35,36^ – hinting again that both routes may bring soluble chemicals into leaf wetness. We note that a rare study on leaf surface organics found mass spectrometry evidence of sugars in dew and frost collected from grass and bush;^37^ likewise, sugars are often listed as the metabolites having the potential to leach through water pores due to their small molecular size.^36,75^ The primarily endogenous origin and the significant content of sugars and carbohydrates also agree with the current understanding of tree-DOM chemistry.^58–60^

A series of additional observations disprove alternative delivery routes and sources. The lack of variation in soak chemistry as a function of stomatal conductance (Table S11†) is a clear sign that endogenous chemicals are not released through open stomata. Likewise, the general decrease in quantitative soak parameters following rain (Fig. 5) reinforces the idea that hydrometeors act as cleansing agents rather than contributing surface matter.^25^ The mass of water-soluble chemicals delivered through dry deposition appears minimal compared to endogenous sources, as indicated by the significant plant-to-plant variability among trees growing within meters of each other's and thus exposed to the same exogenous sources (Fig. 6). Although abundant evidence points to a prevalence of endogenous chemicals, dedicated studies relying on speciated chemical analyses are needed to corroborate this view.

#### Dissolved chemicals in leaf wetness may undergo “rapid” chemistry

Last, we collected a few pieces of evidence supporting the occurrence of “rapid” (*i.e.*, within tens of mins to hours) reactions of soluble organics following their release in water, due to biotic and/or abiotic processes. First, we noticed a depletion of aliphatic compounds for soaking times >15 min (Fig. 4), which we tentatively attributed to consumption by phyllosphere microorganisms. Although we may have artificially sped up this process by handholding (thus, warming) the sampling tubes, the ubiquitous presence of phyllosphere microbes^1^ makes biotic reactions highly probable, especially when water is abundant. Second, we detected minor but consistent differences in organics' quality between soaks and authentic raindrops (Tables 1, S2 and Text S2†). This observation led to the hypothesis that raindrops are more processed than soaks (but less than tree-DOM), possibly due to exposure to atmospheric oxidants and/or microbial processing between on-canopy deposition and collection (generally, 3–5 hours; Table S2†). Remarkably, partial processing of organics has also been hypothesized in the tree-DOM literature – though based on a different set of observations.^59,76^ This difference becomes particularly evident in drops collected after ambient humidity drops below 80%. One can expect these conditions to favor concentration processes and/or condensation reactions,^25^ which is what we observed experimentally (Text S2†). While only preliminary, this data underscores the potential role of transient meteorological conditions as drivers of leaf surface chemistry.^25^

## Conclusions

The surging interest in elucidating multiphase chemistry on plant leaves makes it essential to establish robust sampling methodologies for collecting material from these surfaces. Here, we developed a flexible sampling protocol that mimics natural wetness while minimizing the drawbacks of collecting authentic samples – *i.e.*, reliance on meteorological conditions, prior knowledge of wetness chemistry, limited sample volume, and uncontrolled chemical processing between deposition and collection. Our experiments highlighted a few variables that significantly impact leaf soaks' chemistry and must be controlled while sampling: (1) the ratio of solvent volume-to-leaf area, (2) the solvent-to-leaf contact time, and (3) environmental conditions. Several other methodological details were found to be secondary or irrelevant in impacting bulk chemistry properties, which can be exploited to develop variants of the protocol and compare data across studies.

The data presented in this work also provide new insights into leaf surface processes. We confirmed that a substantial quantity of water-soluble material is available for multiphase reactions on wet leaves (for organics, 0.6–57 μg cm^−2^) and that this amount is highly heterogeneous, both spatially and temporally. Characterizing this heterogeneity across multiple plant species, seasons, and locations is a clear research need. Relying on several lines of evidence, we further hint that organic compounds in ponderosa pine needle soaks and authentic raindrops from the same plant originate from phyllosphere biofilms, the leaf interior (through cuticular water pores), or both – thus, they are primarily endogenous. While still requiring empirical confirmation, this hypothesis may, in part, explain why some atmospheric trace gases display surface reactivity only in the presence of leaf wetness.^7,12,13^ Last, we presented preliminary data supporting the occurrence of “rapid” biotic and/or abiotic processing of organic chemicals after they are released into leaf wetness. These reactions appear to be triggered by changing meteorological conditions (*e.g.*, rapid changes in humidity or temperature) and may represent hot moments for biogeochemical processing at the plant-soil-water continuum.^77^

## Author contributions

R. O. and M. R. conceived the project with contributions from all authors. R. O., C. O., R. K. R., and M. R. collected samples and performed UV-vis, conductivity, and pH measurements. R. O. developed the method for plate reader measurements, carried out TOC and nitrogen analyses, performed most data analysis, and wrote the first draft, which was read and commented by all authors. R. O., M. R., and D. F. acquired funding.

## Conflicts of interest

The authors declare no conflicts of interest.

## Supplementary Material

EM-026-D4EM00065J-s001
