# Supplementary material for: Development of a sampling protocol for collecting leaf surface material for multiphase chemistry studies
Source: Environ Sci Process Impacts. 2024 May 14;26(6):1008–21. doi: 10.1039/d4em00065j (PMC11188671; doi:10.1039/d4em00065j)
Supplement: EM-026-D4EM00065J-s001 [file EM-026-D4EM00065J-s001.pdf]

Electronic Supplementary Material (ESI) for Environmental Science: Processes & Impacts.
This journal is © The Royal Society of Chemistry 2024

Electronic Supplementary Information for

Development of a sampling protocol for the collection of water-soluble material from plant leaves

Rachele Ossola, Rose K. Rossell, Mj Riches, Cameron Osburn, and Delphine Farmer

correspondence to:

rachele.ossola@colostate.edu, mj.riches@colostate.edu

Table of Contents

Text S1: Sampling location and meteorological data ... 3
Overview of meteorological conditions ... 3
Details on ponderosa pines location and leaf morphology ... 5
Text S2: Comparison between needle soaks and authentic raindrops ... 6
Collection and analyses ... 6
Results ... 6
Text S3: Supplementary information on absorbance measurements ... 9
Details on data collection and processing ... 9
Reference solution for 96-well plate measurements ... 11
Spectral parameters calculation ... 12
Text S4: Supplementary methods ... 14
pH measurements ... 14

Portable Photosynthesis System (PPS) 14
Absorbance measurements with the Tecan NanoQuant plate 14
Text S5: Optimization of sample collection and storage 17
Leaching of organics from sampling tubes 17
Pre-leaching of disposable falcon tubes 18
Syringe filtration protocols to limit sample loss 18
Storage containers 18
Storage conditions 19
Text S6: Overview of needle soak chemistry 20
Data source and treatment 20
Analyses of nitrogen species 20
Results 21
Text S7: Details on tests evaluating sampling variables 24
Solvent: Solvent volume-to-leaf area ratio 24
Solvent: Rain vs. MilliQ water 25
Sampling approach: Solvent-to-leaf contact time 26
Sampling approach: Sonication 28
Sampling approach: Filtration 30
Sampling approach: Freezing 31
Leaf: Healthy vs. damaged 32
Leaf: Living vs. dead 33
Leaf: On-plant vs. detached 34
Leaf: Open vs. close stomata 35
Text S8: Details on tests evaluating environmental variables 37
Meteorological conditions 37
Location and plant-to-plant variability 39
References 42

Text S1: Sampling location and meteorological data

Overview of meteorological conditions

Data source and treatment Meteorological data in Fort Collins is from the Fort Collins Weather Station located in the Colorado State University (CSU) Main Campus.[1] This station is ~ 1 km from the Arboretum, ≤ 1 km from other sampling locations on campus, and ~ 10 km from the Horsetooth Mountain Open Space. Data for the CSU Mountain Campus is from the local Main Weather Station.[2] Data on precipitation (inch or mm), ambient relative humidity (RH; %), solar radiation (W m^{-2}), and air temperature (˚F or ˚C) were downloaded from the web interface as 1-h averages,[3,4] converted to metric units, and processed for selected time periods to yield cumulative precipitation (V_{rain}), average RH (RH_{avg}), average solar radiation (I_{avg}), average air temperature (T_{avg}), and approximate time after last rain > 0.5 mm (Δt_{rain}). For calculating this last parameter, we assumed that sampling always took place at 9:00 AM.

Results The weather in Fort Collins was overall dry from January until May 9, 2023, except for two intense rain events on April 4 and 5 (168 and 90 mm, respectively; Figure S1A). May 9 until June 17 was wet, with 350 mm of rain; of these, 172 mm fell during the sampling campaign in three major and two minor events (Figure S1B). The three main events occurred on June 1 – 5 (V_{rain} = 80 mm, RH_{avg} = 83%, I_{avg} = 180 W m^{-2}, T_{avg} = 15 ˚C), June 11 – 13 (V_{rain} = 42 mm, RH_{avg} = 84%, I_{avg} = 150 W m^{-2}, T_{avg} = 15 ˚C), and June 15 – 17 (rain = 39 mm, RH_{avg} = 81%, I_{avg} = 200 W m^{-2}, T_{avg} = 15 ˚C). From June 18 until end of sampling, the weather was dry and summer-like, with < 0.5 mm of rain, an average RH of 56%, average radiation intensity of 316 W m^{-2}, and an average air temperature of 21˚C.

Tests to investigate the role of sampling variables (Text S7) were performed over five days between May and June 2023 (Figure S1B, circles). May 22 was clear in the morning and partly overcast in the afternoon, with RH_{avg} = 52%, I_{avg} = 273 W m^{-2}, T_{avg} = 18 ˚C, and Δt_{rain} = 81 h. June 6 was a clear sunny day with minor cloud cover in the late afternoon, and having RH_{avg} = 70%, I_{avg} = 343 W m^{-2}, T_{avg} = 18 ˚C, and Δt_{rain} = 14 h. The ambient relative humidity was > 80% until 8:00 AM due to recent precipitation. June 8 was a clear sunny day with RH_{avg} = 54%, I_{avg} = 338 W m^{-2}, T_{avg} = 20 ˚C, and Δt_{rain} = 62 h. June 21 was partially overcast and humid, with light rain in

the evening (RH_{avg} = 70%, I_{avg} = 234 W m^{-2}, T_{avg} = 20 ˚C, Δt_{rain} = 91 h). June 26 was a clear sunny day, with RH_{avg} = 59%, I_{avg} = 365 W m^{-2}, T_{avg} = 22 ˚C, and Δt_{rain} = 211 h.

Tests investigating the role of meteorological conditions (Text S8) took place between June 8 and June 27 in the Arboretum (light orange line in Figure S1B). The weather was wet until June 17 (with two major and one minor rain event), then changed to summer-like conditions (details in the main text). The "Location and plant-to-plant variability" test (Text S8) was carried out on June 18 and 19. On June 18, we took samples at the CSU Mountain Campus over an overcast day (RH_{avg} = 41%, T_{avg} = 13 ˚C, Δt_{rain} = 114 h; the overall data for this location in June were 9.1 mm of rain, RH_{avg} = 64%, and T_{avg} = 9.8 ˚C). On June 19, we visited the Horsetooth Mountain Open Space in the morning and the CSU Main Campus in the afternoon. The day was warm and sunny, with RH_{avg} = 49%, I_{avg} = 369 W m^{-2}, T_{avg} = 23 ˚C, and Δt_{rain} = 43 h (dark diamond in Figure S1B).

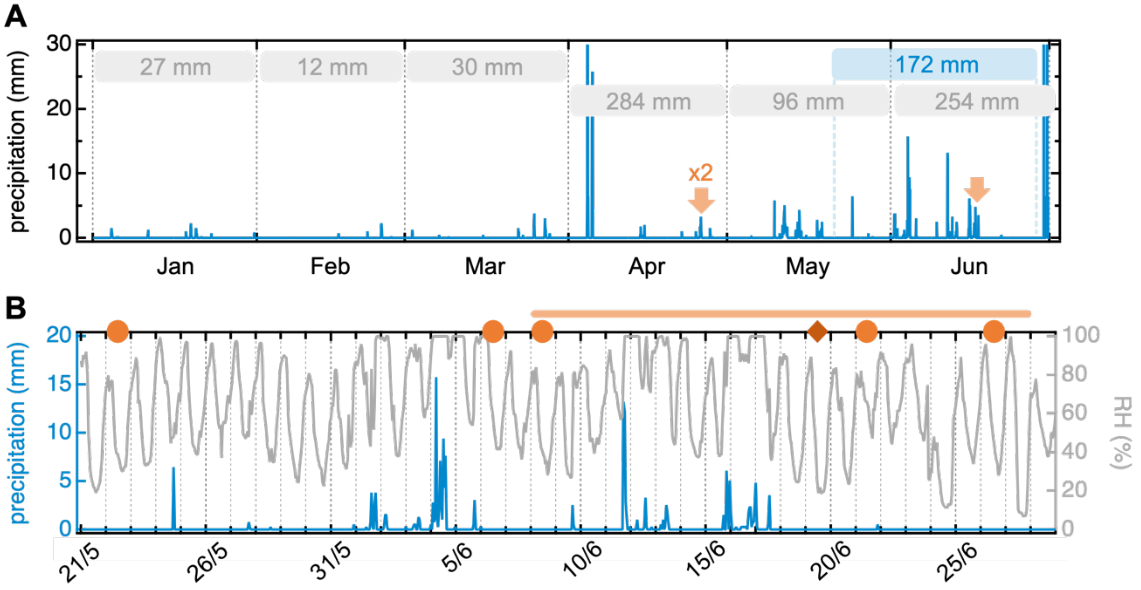

Figure S1 A Precipitation in Fort Collins (CO, US) in the first semester of 2023. Grey numbers are cumulative monthly amounts; cumulative precipitation during the sampling period (May 22 to June 27) is indicated in blue. Orange arrows denote days of raindrop collection. **B** Zoomed view of panel **A** for May 21 to June 28 overlayed with ambient RH data (grey). Orange circles identify collection days during which we tested the role of sampling variables (Text S7). The light orange line identifies days during which we investigated the effect of meteorological conditions; the orange square marks the day used for location and plant-to-plant variability tests (only Horsetooth Mountain Open Space and CSU Main Campus; Text S8).

Details on ponderosa pines location and leaf morphology

Location	GPS coordinates	Tree		Needle	
		C_{BH} (in)	Approx. age (yr)	Length (cm)	Diameter (cm)
CSU Arboretum	40.5708 N, 105.0916 W	48	76	18.8 ± 2.5†	0.155 ± 0.011†
CSU Main Campus	40.5751 N, 105.0913 W	60	95	11.5 ± 1.4	0.16 *
CSU Main Campus	40.5736 N, 105.0876 W	102	161	17.0 ± 0.6	0.172 ± 0.015
CSU Main Campus	40.5752 N, 105.0854 W	75	118	13.6 ± 0.8	0.179 ± 0.017
CSU Main Campus	40.5714 N, 105.0854 W	83	131	14.4 ± 1.4	0.192 ± 0.11
Horsetooth Mountain Open Space	40.5302 N, 105.1844 W	56	88	15.2 ± 1.2	0.162 ± 0.006
Horsetooth Mountain Open Space	40.5301 N, 105.1846 W	46	73	13.0 ± 2.7	0.17 ± 0.03
Horsetooth Mountain Open Space	40.5337 N, 105.1925 W	66	104	15.1 ± 1.1	0.19 ± 0.03
Horsetooth Mountain Open Space	40.5338 N, 105.1925 W	78	123	12.7 ± 0.9	0.149 ± 0.010
CSU Mountain Campus	40.56636 N, 105.55460 W	81	128	12.4 ± 1.7	0.22 ± 0.02
CSU Mountain Campus	40.56650 N, 105.55460 W	67	106	13.4 ± 0.9	0.165 ± 0.006
CSU Mountain Campus	40.56486 N, 105.55557 W	82	129	11.8 ± 1.2	0.205 ± 0.006
CSU Mountain Campus	40.56542 N, 105.55557 W	101	159	11.4 ± 0.3	0.165 ± 0.011

Table S1 Location and main morphological data for *Pinus ponderosa* specimens. Tree age was approximated from the circumference at breast height (C_{BH}, in in) as $C_{\mathrm{BH}}/(\pi \cdot v_{\mathrm{DBH}})$, where v_{DBH} is the diameter growth rate (in in yr^{-1}). As growth rates for this species are highly variable, the reported value is the average between the age obtained with $v_{\mathrm{DBH}}^{max} = 0.87$ in yr^{-1} (productive site) and $v_{\mathrm{DBH}}^{min} = 0.114$ in yr^{-1} (non-productive site).[5] Needle length and diameter are averages of three values obtained with a ruler and a caliper, respectively, and the error is their standard deviation. † Average of 35 measurements instead of three. * All needles had the same diameter, so the standard deviation is negligible.

Text S2: Comparison between needle soaks and authentic raindrops

Collection and analyses

Raindrops were collected shortly after the end of three rain events from the needles of the ponderosa pine in the Arboretum, either as composite samples (i.e., 50 to 60 drops) or individual drops (Table S2). In all cases, drops gathered at the needle tips were gently rolled into a new 1.5 mL Eppendorf tube (Eppendorf North America, 022364111). Samples were analyzed immediately after collection or frozen until analyses. We used undiluted aliquots for pH, UV-vis absorbance, and some conductivity measurements. Due to volume limitations, we diluted some samples for conductivity measurements (10-fold) and all samples for TOC and total nitrogen (TN) analyses (66- to 100-fold). Absorbance measurements were performed with a Spark Tecan multimode microplate reader equipped with a Tecan NanoQuant plate as detailed in Text S4 and Figure S4. Total nitrogen analysis is described in Text S6.

Results

Composite samples. Overall, raindrops collected from ponderosa pine needles contain significant concentrations of organic and inorganic species, with [TOC] = 55 – 180 $mg_C\ L^{-1}$, $\Sigma\alpha_{200-400}$ = 230 – 530 cm^{-1}, and conductivity between 70 and 400 $\mu S\ cm^{-1}$ (Table S2). These concentrations are more than an order of magnitude higher than needle soaks (Table S3) and, for organic carbon, comparable to tree-DOM.[6,7] This result may be justified by higher solvent volume-to-leaf area of raindrops vs. soaks or be due to raindrops being continuously in contact with areas of dry cuticles as they drip downwards, which makes them increasingly more concentrated.

Quantitative chemistry parameters are comparable, although not identical, to needle soaks (Table 1), hinting that water-soluble organics are overall similar for collected raindrops and needle soaks. We note the slight but not significant increase in $SUVA_{254}$ and decrease in $S_{275-295}$ for raindrops as compared to soaks. Remarkably, tree-DOM has even higher $SUVA_{254}$ (1.6 – 2.8 $L\ mg_C^{-1}\ m^{-1}$ for throughfall) and lower spectra slope (< 0.017 nm^{-1}) than authentic raindrops,[6–8] leading to the hypothesis of chemical processing between deposition and collection. This process may be at its initial steps in raindrops collected from tree crowns and in a more advanced stage in throughfall, while 5-min soaks may effectively represent “time zero” leachates.

Individual raindrops. During one of the rain events, we collected both composite and individual raindrops. Most samples were taken 5 h after the end of rain (samples 3 and 3a-g), when the tree was still wet and ambient RH was above 80%. A single drop (sample 4) was also collected 1.5 h later, after ambient relative humidity dropped below 80%. As single raindrops ranged from 7 – 20 μL in volume, we performed only absorbance measurements.

We observed a consistent variability in $\Sigma\alpha_{200-400}$ among raindrops collected at the same time, with values ranging from 4 to 440 cm^{-1} and a median of 44 cm^{-1} (Table S2). The corresponding composite sample had an integrated absorbance of 247 cm^{-1}, considerably higher than the median of individual raindrops. This large variability and the key impact of a few outliers match well with our observations for needle soaks. When converted to TOC values (see Table S2 caption for details), the estimated carbon content of individual raindrops ranges from 1.5 to 150 $mg_C\ L^{-1}$.

Spectral parameters of individual raindrops were consistent and comparable to the composite sample collected at the same time. We observed again a single outlier with an absorption spectrum more like pristine rain ($\Sigma\alpha_{200-400}$ = 4.0 cm^{-1}, $\alpha_{215}/\alpha_{200}$ = 0.55; Table S4) than needle-collected drops (sample 3a; Table S2). This variability underlines the need for sample replicates also when collecting raindrops.

Sample 4 (collected at RH < 80%) represents a unicum due to its extreme concentration, which we estimated in the g L^{-1} range (Table S2). This raindrop was also visibly colored (yellow), a feature that has been reported in dew and frost samples collected from leaf blades.[9] Although we cannot form reliable conclusions from a single datapoint, this sample hints that water evaporation may drive changes in concentration and/or chemistry in raindrops sitting on plant leaves.

ID	Date	Time after rain	[TOC] (mg_C L^{-1})	[TN] (mg_N L^{-1})	$\Sigma\alpha_{200-400}$ (cm^{-1})	Cond (µS cm^{-1})	pH	$\alpha_{215}/\alpha_{200}$	$\alpha_{260}/\alpha_{200}$	$SUVA_{254}$ (L mg_C^{-1} m^{-1})	$S_{275\text{-}295}$ (nm^{-1})
**Composite samples**											
1	25/04	4 h	178	36	533	396	6.7	0.60	0.15	1.5	0.019
2	26/04	3 h	56	16	226^{b}	91	7.0	0.55^{b}	0.26^{b}	2.8^{b}	0.022^{b}
3	26/04	5 h	109	8	247	153	6.5	0.62	0.20	1.4	0.019
5	16/06	0 h	104	*(2)*^{a}	259	72	5.8	0.63	0.20	1.4	0.020
**Individual drops**											
3a	26/04	5 h	(≈ 1.5)^{c}		4^{d}			0.53^{d}	– 0.02^{d}		n.c.^{d}
3b	26/04	5 h	(≈ 10)^{c}		29			0.53	0.14		0.019
3c	26/04	5 h	(≈ 150)^{c}		442			0.61	0.22		0.017
3d	26/04	5 h	(≈ 20)^{c}		58			0.55	0.14		0.013
3f	26/04	5 h	(≈ 30)^{c}		80			0.58	0.17		0.020
3g	26/04	5 h	(≈ 10)^{c}		24			0.58	0.14		0.014
4	26/04	6.5 h*	(≫ 900)^{c}		≫ 2400^{d}			n.c.^{d}	n.c.^{d}		0.019^{d}
Average (excluding 2, 3a, and 4)								0.59 ± 0.04	0.17 ± 0.03	1.40 ± 0.06	0.018 ± 0.003

Table S2 Quantitative and qualitative parameters for authentic raindrops collected from ponderosa pine needles. Total nitrogen analyses are described in Text S6. Time after rain is estimated from meteorological data from the Fort Collins Weather Station (Text S1 and Figure S1); in all cases but sample 4 (marked with an asterisk), RH was above 80% at the time of collection. For sample 4, RH dropped below 80% 1.5 hours before sample collection. [a] Data < LoQ. [b] The absorption spectrum had a feature at 245 nm. [c] Estimated as [TOC] = 0.35 $\Sigma\alpha_{200-400}$, which was defined empirically using data from composite samples ($R^2 = 0.98$; intercept was forced to zero). [d] The absorption spectrum was more comparable to pure rainwater than needle-collected raindrops. As $\alpha \approx 0$ above 235 nm, $\alpha_{260}/\alpha_{200}$ and $S_{275\text{-}295}$ could not be calculated (n.c.). [e] The absorption spectrum saturated below 225 nm – thus, $\Sigma\alpha_{200-400}$ was estimated as being larger than 2400 cm^{-1} and absorption coefficient ratios were not calculated (n.c.).

Text S3: Supplementary information on absorbance measurements

Details on data collection and processing

General measurement setup. Each run included at least three MilliQ water blanks, at least one Suwannee River natural organic matter reference sample (SRNOM; details below), and the analytes (≤ 91 samples; Figure S2, top). For ≥ 91 samples, we prepared two or more 96-well plates, each with their separate blanks and references.

Data analysis protocol. Data from each run was analyzed following the procedure outlined in Figure S2 (bottom). First, we calculated a single, average blank spectrum ($\overline{A_{blank}}$). As we measured spectra in triplicate with at least 3 blanks per run, $\overline{A_{blank}}$ was the average of at least 9 individual vectors. Second, for each analyte and reference sample, we obtained the average raw spectrum ($\overline{A_{raw}}$) as the mean of the three measurement replicates. Third, we corrected the average raw spectrum for blank absorption as $\overline{A_{corr,blank}} = \overline{A_{raw}} - \overline{A_{blank}}$. Fourth, we calculated the offset (Ω_A) of each spectrum as the average $\overline{A_{corr,blank}}$ between 700 and 800 nm, a wavelength region where these samples do not absorb. Fifth, we corrected $\overline{A_{corr,blank}}$ for the offset, yielding $\overline{A_{corr}} = \overline{A_{corr,blank}} - \Omega_A$. Sixth, we used the corrected SRNOM absorption spectrum to compute the run's pathlength (ℓ, in cm) as detailed below. When we had more than one reference sample per run, we first calculated an average $\overline{A_{corr}}$ for SRNOM and used this spectrum for pathlength calculation. Seventh, we converted absorption values into decadic absorption coefficients (α, in cm^{-1}) by dividing the corrected absorption by the pathlength ($\alpha = \overline{A_{corr}}/\ell$). We note that measuring spectra in triplicate was not strictly necessary with the Spark Tecan multimode microplate reader (average relative error ≤ 2% between 200 and 800 nm) but was crucial with an older instrument we used initially for method development (Tecan Infinite M200).

Pathlength calculation. ℓ of each individual run was obtained from the slope of the plot in Figure S3B – where the y-axis is $\overline{A_{corr}}$ for SRNOM and the x-axis is the absorption spectrum of the exact same solution measured with a bench-top UV-vis spectrophotometer (Cary 3500 Compact UV-Vis Spectrophotometer) and a 1-cm pathlength quartz cuvette. The benchtop instrument was run in double-beam mode with 1 nm wavelength increments and MilliQ water as reference. Spectra recorded with this instrument were only corrected for blank absorption and offset (steps 3 – 5 in Figure S2). For pathlength calculation, we generally excluded data < 215 – 230 nm, as they

deviated from linearity. This is the same spectral region that we identified as susceptible to contamination based on repeated SRNOM absorption measurements and MilliQ water blanks (Figure S3A). Overall, we observed minimal variations in ℓ across all runs (range: 0.52 to 0.62 cm; mean: 0.58 ± 0.03 cm; N = 12), which we attributed to slight changes in solution volume and/or ambient temperature.

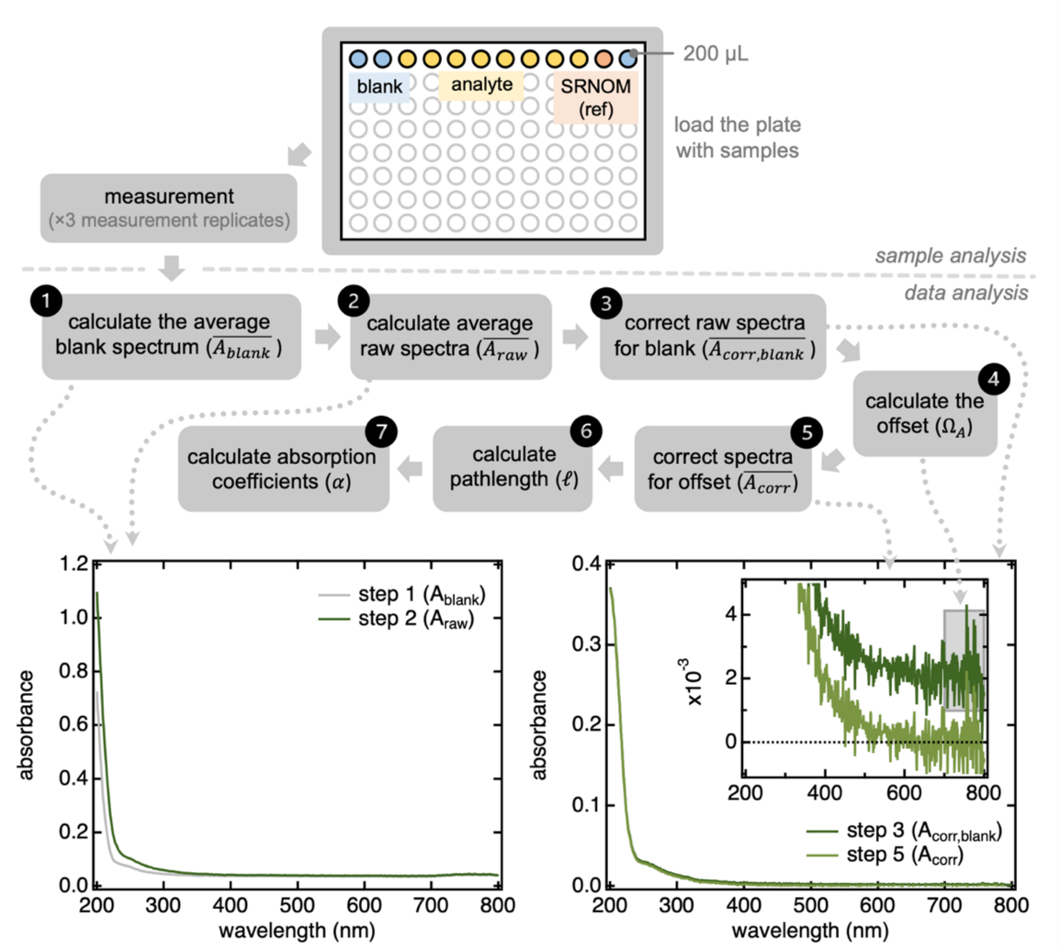

Figure S2 Workflow for data collection (top) and analysis (bottom) using a plate reader equipped with a UV-Star 96-well microplate. All runs included at least three MilliQ water blanks (light blue) and one reference sample (orange) in addition to the analytes (yellow). The two example outputs are for $\overline{A_{blank}}$ (step 1; gray trace), $\overline{A_{raw}}$ (dark green trace on the left graph; step 2), $\overline{A_{corr,blank}}$ (dark green trace on the right graph; step 3), Ω_A (gray square in insert; step 4), and $\overline{A_{corr}}$ (light green trace; step 5). The insert in the right graph is a zoomed view of the main plot at low absorbance values. Data represented in the plots is one replicate of the open stomata test. The offset calculated using data shown in the insert was 0.0021 ± 0.0007 before correction (dark green trace) and 0.0000 ± 0.0007 after correction (light green trace).

Reference solution for 96-well plate measurements

As needle soaks are complex mixtures, we used a commercial dissolved organic matter sample as the reference sample for UV-Vis analyses. The reference solution was prepared upon dilution of a concentrated Suwannee River natural organic matter stock (SRNOM; 2R101N, International Humic Standard Society) in MilliQ water, and was stored in the fridge (4˚C) for several months in the original volumetric flask (100 mL, 1.8 mg_C L^{-1}). The concentrated stock (≈ 200 mg_C L^{-1} in MilliQ water) was prepared according to published protocols.[10]

The reference solution's absorption spectrum was highly reproducible across the ~ 6 months of the projects, with relative errors of 5–18% between 200 and 400 nm (Figure S3A; $N = 12$). Below 230 nm, the relative error increases slightly due to organic carbon contamination (which we commonly observed in MilliQ water blanks; insert in Figure S3A), while above 400 nm, it spikes due to the sharp decrease in absorbance. The average spectrum in Figure 3A has $\alpha_{215}/\alpha_{200} = 0.84 \pm 0.04$, $\alpha_{260}/\alpha_{200} = 0.46 \pm 0.06$, $SUVA_{254}$ = 4.2 L mg_C^{-1} m^{-1}, and $S_{275\text{-}295} = (0.014 \pm 0.001)$ nm^{-1} (Table 1, main text).

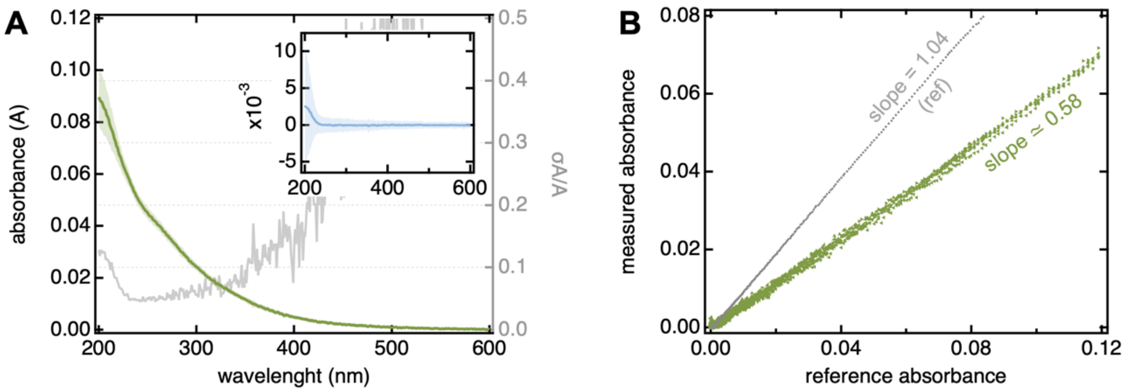

Figure S3 **A** Average UV-Vis spectrum of the SRNOM reference solution measured with a Spark Tecan plate reader and a UV-Star 96-well plate (200 µL; average of 12 spectra). Data was processed as outlined in Figure S2 but without pathlength correction. The green shade is the standard deviation (σ_A), while the grey data is the relative error (σ_A/A). The insert shows the average instrument blank absorbance and its standard deviation (200 µL; average of 47 spectra). **B** Plot used for pathlength determination (step 6 in Figure S2). The x-axis is the SRNOM absorption spectrum obtained with a benchtop spectrophotometer and a 1-cm quartz cuvette. The grey datapoints are acquired with the same benchtop instrument and solution but on a different day and are reported only for reference.

Spectral parameters calculation

After data workup, we processed all absorption spectra to obtain quantitative and qualitative metrics. As quantitative parameter (i.e., theoretically proportional to organic carbon concentrations), we integrated the decadic absorption coefficient between 200 and 400 nm to yield $\Sigma\alpha_{200-400}$ (eq S1; in cm^{-1}).

$$\Sigma\alpha_{200-400} = \sum_{200}^{400} \alpha_\lambda \qquad \text{S1}$$

This metric is conceptually analogous to the absorption coefficient at 254 nm (α_{254}), which is a common proxy for organic carbon concentrations in aquatic chemistry studies.[11,12] For soaks, we deemed $\Sigma\alpha_{200-400}$ more appropriate than α_{254} due to the typically low absorption (and, consequently, high relative error) above 250 nm (see Figure 3A).

As qualitative metrics, we used (1) the ratio of absorption coefficients at 215 and 200 nm ($\alpha_{215}/\alpha_{200}$); (2) the ratio of absorption coefficients at 260 and 200 nm ($\alpha_{260}/\alpha_{200}$); (3) the specific ultraviolet absorbance at 254 nm ($SUVA_{254}$, in L mg_C^{-1} m^{-1}); and (4) the spectral slope between 275 and 295 nm ($S_{275\text{-}295}$, in nm^{-1}). The first two are inspired by E2/E3, the ratio of a sample's absorption at 254 nm (or 250 nm) to that at 365 nm.[12] This parameter is commonly used in aquatic chemistry studies as a proxy for dissolved organic matter's (DOM) average molecular weight and aromaticity[11,12] but we found it inappropriate for soaks due to the poor signal-to-noise ratio above 300 nm. Instead, we selected $\alpha_{215}/\alpha_{200}$ and $\alpha_{260}/\alpha_{200}$ as metrics for the relative amount of the two pools of chromophores detected in every soak (above and below 230 nm, respectively). $SUVA_{254}$ and $S_{275\text{-}295}$, are commonly employed in DOM studies as proxies for aromaticity and average molecular weight, respectively.[11–14] The first is calculated as

$$SUVA_{254} = \frac{\alpha_{254} \cdot 10^2}{[TOC]}, \qquad \text{S2}$$

where α_{254} is the decadic absorption coefficient at 254 nm (in cm^{-1}), [TOC] is the total organic carbon concentration (in mg_C L^{-1}), and 10^2 is a conversion factor from cm^{-1} to m^{-1}.[14] $SUVA_{254}$ is inversely proportional to the percent of aromatic carbon measured via ^{13}C-NMR and, in DOM samples, it ranges from ~ 0.5 L mg_C^{-1} m^{-1} (open ocean fulvic acid, 8% aromatic carbon) to ~ 5 L mg_C^{-1} m^{-1} (terrestrial humic acids, 40% aromatic carbon).[14,15] Due to the strong dependency on

TOC, we excluded individual $SUVA_{254}$ values from average calculations in samples with TOC < LoQ. To obtain $S_{275-295}$, we plotted the natural log of the decadic absorption coefficient (ln α) vs. wavelength and we fitted the data between 275 and 295 nm to a line, yielding $S_{275-295}$ = – slope.[13] In DOM samples, $S_{275-295}$ is inversely proportional to the average molecular weight, and it ranges from 0.009 nm^{-1} (4000 Dalton) to > 0.020 nm^{-1} (< 1000 Dalton).[13] We note that $S_{275-295}$ may be unreliable in the presence of discrete absorption features, as we observed in two ponderosa pines of the unmanaged urban site (see Table S12 and main text).

Text S4: Supplementary methods

pH measurements

pH measurements were performed with a compact pH meter (LAQUAtwin-pH-33, Horiba Scientific). The sensor was first conditioned with a pH 7 buffer for at least three hours and then calibrated with commercial buffers at pH 4 (Millipore Sigma, BX1628-1), 7 (Fisher Chemicals, SB107-500), and 10 (Millipore Sigma, BX1633-1). The sensor was washed with MilliQ water, wiped gently, and rinsed with 60 μL of analyte. We then placed an additional 120 μL of analyte, closed the lid, and waited for stabilization. We report pH as the average of three stable values obtained by opening and closing the lid three times ($\sigma_{\mathrm{pH}} = \pm 0.03$ on average, with values up to ± 0.1). Every 5 – 10 samples, we measured a pH 7 buffer aliquot to assess precision (0.2 – 0.8%) and accuracy (0.01 – 2.5%). All solutions were allowed to equilibrate to room temperature before analysis. Due to unstable readings, we only analyzed samples above background conductivity.

Portable Photosynthesis System (PPS)

Stomatal conductance and CO_2 assimilation were determined using a portable photosynthesis system (LI-6800, Li-Cor Intl.) as detailed in Riches et al.[16] Briefly, 10 needles were arranged in a flat layer and clamped into the leaf chamber, which maintained leaf-level CO_2 at 415 μmol mol^{-1}, relative humidity at 75%, and air temperature at 27 °C in a well-circulated airspace. Measurements to verify stomatal closure following dark adaptation were conducted without light.

Absorbance measurements with the Tecan NanoQuant plate

Raindrops were analyzed with the Spark Tecan microplate reader equipped with a Tecan NanoQuant plate, a quartz microplate designed for low-volume samples. Figure S4 gives an overview of the optimized workflow for data collection; data analysis was carried out as in Figure S2 with the three differences outlined below.

Collection parameters. The plate reader was programed to read absorption values as single measurement replicates in the 200 – 800 nm wavelength range with 1-nm increments.

Data collection. Before depositing the samples, the plate was cleaned thoroughly with ethanol and deionized water. This step was best performed by wetting a Kimwipe or a piece of lens paper with

the solvent and passing it gently on all surfaces, including the lenses on the outside of the plate. To confirm that cleaning was successful, we deposited 2 µL of MilliQ water on each well (using a calibrated 1 – 10 µL pipettor), collected absorption spectra, and inspected for large differences among positions. We iterated these steps until all wells showed the same spectrum.

After cleaning, we loaded the plate with MilliQ water and collected the first series of blanks. We dried the inner surfaces with a piece of lens paper and loaded the first set of four analytes (2 µL onto 4 nearby wells, for a total of 8 µL per analyte; Figure S4). After measurement, we cleaned the plate and loaded a new set of samples. In this case, we absorbed the liquid with a Kimwipe and passed a piece of lens paper wetted with MilliQ water on all internal surfaces; all surfaces were then dried with a clean piece of lens paper. In the last iteration, we loaded a concentrated SRNOM reference solution (~20 $mg_C\ L^{-1}$, prepared upon dilution of the same stock used for the 96-well plate reference) and collected its absorption spectrum. The last run was always an additional series of blanks.

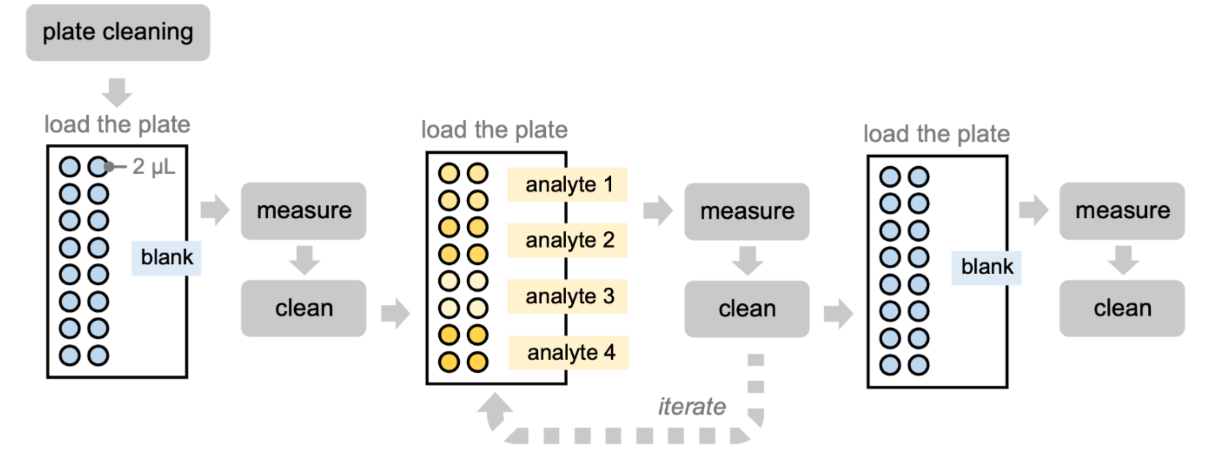

Figure S4 Workflow for data collection using the Tecan NanoQuant plate and a Spark Tecan microplate reader. The central step was iterated until all analytes were measured; the last iteration always includes a SRNOM reference sample (≈ 20 $mg_C\ L^{-1}$), which is needed pathlength calculation. Blanks were measured at the beginning and the end.

Data analysis. Data from each series of runs was analyzed following the general procedure in Figure S2 but with three key differences. First, all samples and measurement replicates were used to obtain $\overline{A_{blank}}$ (step 1) – thus, $\overline{A_{blank}}$ was the average of 16 × 2 = 32 individual vectors. Second,

$\overline{A_{raw}}$ (step 2) was the mean of the four *sample*, not measurement, replicates. Lastly, we used a more concentrated SRNOM reference solution because the 96-well plate reference sample was too dilute. For this plate, we obtained an average pathlength of (0.0474 ± 0.0009) cm ($N = 3$).

Text S5: Optimization of sample collection and storage

Leaching of organics from sampling tubes

Experiment. New disposable falcon (15 mL, for soaks) and Eppendorf tubes (1.5 mL, for raindrops) were filled with MilliQ water and left at room temperature, in the fridge (4˚C; only Eppendorf tubes), or in the freezer (– 10˚C; only Eppendorf tubes) for up to 2 months. Periodically, we withdrew aliquots for UV-Vis and conductivity analyses. We also performed TOC analyses on selected datapoints.

Results. UV-Vis analyses showed clear evidence of organic carbon contamination in new falcon tubes ($\Sigma\alpha_{200-400}$ up to 13 cm^{-1}; Figure S5A-B), while the average $\Sigma\alpha_{200-400}$ value for Eppendorf tubes was always low (Figure S5C) and overall comparable to the instrument blank ((0.08 ± 0.34) cm^{-1}, N = 47). Conductivity was always indistinguishable from MilliQ water, indicating no measurable contamination from inorganic ions. We confirmed organic carbon contamination in new falcon tubes via TOC analyses, finding that MilliQ water contained 0.7 and 2.8 $mg_C\ L^{-1}$ after 14 and 56 days, respectively, of storage in these containers. The absorption spectrum of these contaminants has absorption features at 229 and 256 nm (Figure S5B), two bands that we never observed in experimental needle soaks.

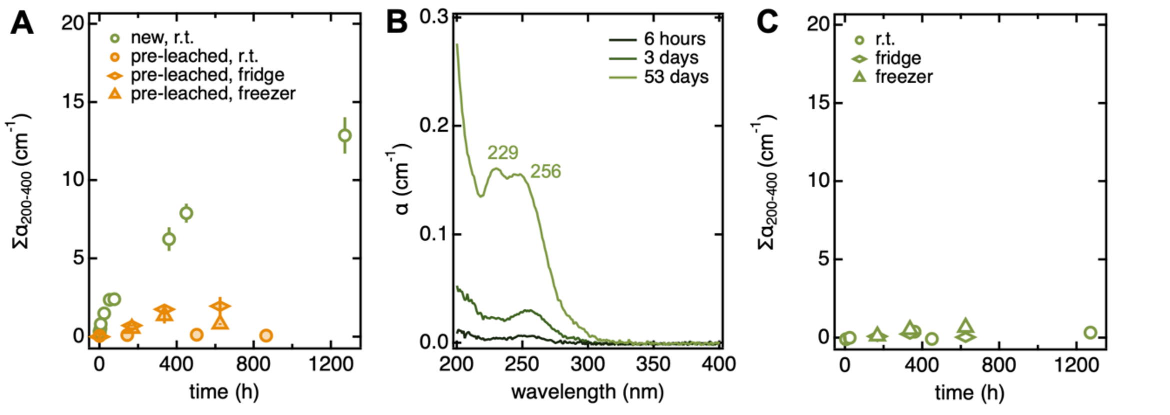

Figure S5 Leaching of organic contaminants from new (green) and pre-leached (orange) falcon tubes (**A**) and new Eppendorf tubes (**C**) kept at room temperature (r.t.; circles), in the fridge (diamonds), or in the freezer (triangles). Panel **B** shows the UV-Vis spectrum of MilliQ water kept in a new falcon tube for up to 53 days. Datapoints in panels **A** and **C** are averages of two to four replicates and error bars are their standard deviation (within the symbol when not visible).

Pre-leaching of disposable falcon tubes

Procedure. New disposable falcon tubes were filled completely with MilliQ water and left at room temperature for at least 3 days. The water was then discarded, then the tubes were rinsed thoroughly with new MilliQ water, left upside down to dry, capped and stored in a clean bag until use.

Testing. We tested the pre-leached tubes with the same procedure described above and found negligible contamination after one month (Figure S5A). Experimental blanks (N = 5) prepared at the time of sample collection and analyzed alongside the samples confirmed that pre-leaching minimizes, but does not completely remove, organic carbon contamination ($\Sigma\alpha_{200-400}$ = (0.7 ± 0.3) cm^{-1}; [TOC] = (0.07 ± 0.05) mg_C L^{-1}, always < LoD). We note that this conclusion is valid for short to medium contact times (i.e., < 1 month).

Syringe filtration protocols to limit sample loss

Experiment 13 mm and 30 mm 0.22 µm polytetrafluoroethylene hydrophilic syringe filters (Tisch Environmental, SF18238 and SF18249) were mounted on disposable 10 mL plastic syringes (Fisher Scientific, 14955459). We first "primed" the filter by pushing through up to 50 mL of MilliQ water and collecting several intermediate aliquots. We then "rinsed" the filter by pushing through up to 3 mL of SRNOM reference solution (1.8 mg_C L^{-1}), taking again several 0.5 mL aliquots. All aliquots were analyzed via UV-Vis spectroscopy to identify minimum priming and rinsing volumes – that is, the volumes at which the aliquot's $\Sigma\alpha_{200-400}$ value is comparable to that of unfiltered MilliQ water ((0.08 ± 0.34) cm^{-1}) and SRNOM ((10.8 ± 0.2) cm^{-1}), respectively.

Results For the 13 mm filter, priming and rinsing require comparable volumes (≥ 0.4 and ≥ 0.5 mL, respectively), while, for the 30 mm filter, priming needs considerably more volume (10 – 15 mL) than rinsing (≥ 1.5 mL). Thus, we recommend priming directly with the sample (≥ 0.5 mL) if using 13 mm syringe filters. If employing 30 mm filters, we suggest to first prime the filter with > 15 mL of MilliQ water and then rinse it with > 1.5 mL of sample.

Storage containers

Experiment New and acid-washed Nalgene bottles were filled with MilliQ water and left in the fridge for up to 9 months (this test was started in the context of a previous project; the procedure

for acid-washing is described in the main text). After this period, we measured the MilliQ water absorption spectrum using the plate reader or the benchtop spectrophotometer.

Results Absorbance measurements showed no detectable organic carbon contamination in any of the treatment, with $\Sigma\alpha_{200-400}$ = (0.27 ± 0.30) cm^{-1} after 9 months (N = 3, all treatments considered).

Storage conditions

Experiment We collected five 20-needle soak replicates from the ponderosa pine in the Arboretum (10 mL of MilliQ water for 5 min). The replicates were combined into a single container to yield 50 mL of soak. Then, 25 mL were filtered through a 30 mm syringe filter (previously primed with 15 mL of MilliQ water and rinsed with 2 mL of sample) into two acid-washed 60 mL Nalgene bottles ("filtered"). The remaining 25 mL were aliquoted into two additional 60 mL Nalgene bottles ("unfiltered"). Filtered and unfiltered subsamples were stored in either the fridge or freezer for up to two months. Periodically, we performed pH, conductivity, and absorbance measurements for a total of four to five datapoints. Samples were allowed to thaw and warm to room temperature before analyses.

Results None of the treatments showed more than 5 – 10% change in the measured parameters during the 2-month storage period. Specifically, pH was within – 1% to +6% of the initial value, conductivity was within +1% to +7%, $\Sigma\alpha_{200-400}$ was within – 1% to +7%, $\alpha_{215}/\alpha_{200}$ was within – 0.5% to +2%, $\alpha_{260}/\alpha_{200}$ was within – 2% to +7%, and $S_{275\text{-}295}$ was within – 2% and + 5%. We noticed some trends in absorption parameters as a function of time, but changes were always within 10% of the initial value – thus, comparable to typical measurement errors.

Text S6: Overview of needle soak chemistry

Data source and treatment

This analysis is based only on 20-needle soaks obtained from the ponderosa pine in the Arboretum between May 22 and June 27, all of which are prepared by immersing the tips of 20 living needles (total submerged area of ≈ 53 cm^2) in 10 mL of MilliQ water for 5 min. Samples were collected in the contexts of various tests – namely, three samples are from the "Rain vs. MilliQ" test (MilliQ water controls only), five samples are from the "Solvent-to-leaf contact time" test (5 min soaks only), and twenty samples are from the "Meteorological condition" test (see Text S7).

Data for these 28 samples were pooled together to evaluate average (± standard deviation), median, and min-max ranges for TOC, conductivity, $\Sigma\alpha_{200-400}$, pH, total nitrogen, ammonium, nitrate, and total organic nitrogen (Table S3). We additionally calculated average (± standard deviation) of all spectral parameters (Table 1, main text) and produced an average absorption spectrum (Figure 3, main text). Last, we plotted each quantitative parameter against conductivity and $\Sigma\alpha_{200-400}$ (Table S3 and Figure S6).

Analyses of nitrogen species

Total nitrogen (TN) was quantified with a Shimadzu TOC-L analyzer during TOC analyses (Shimadzu Scientific Instruments, Inc.). 100 µL of samples were injected in duplicate or triplicate with sparge flow of 80 and sparge time of 1:30 min. LoD and LoQ were 0.02 – 0.06 mg_N L^{-1} and 0.06 – 0.20 mg_N $L^{-1,}$ respectively (ranges refer to different analysis runs). We note that these analyses were performed on diluted samples; while dilution was not a problem for TOC, most non-blank samples were < LoQ and up to 6% of all samples were < LoD. For the 28 samples considered here, 4 were < LoQ (14%) but all were above the LoD.

Ammonium and nitrate were measured with a continuous flow analyzer (Alpkem Flow Solution IV; O.I. Analytical, College Station, Texas) following standard methods (EPA #353.2 and DIN #38406 for nitrate and ammonium, respectively). Nitrate had $LoD_{NO_3^-}$ = 0.001 – 0.007 mg_N L^{-1} and $LoQ_{NO_3^-}$ = 0.003 – 0.022 mg_N L^{-1}. For ammonium, experimental blanks were considerably higher than instrumental ones ((0.048 ± 0.016) vs. – (0.0051 ± 0.0053) mg_N L^{-1}) – thus, here we obtained LoD and LoQ from the standard deviation of experimental rather than instrumental blanks

($LoD_{NH_4^+}$ = 0.05 mg$_N$ L^{-1}, and $LoQ_{NH_4^+}$ = 0.16 mg$_N$ L^{-1}). Like TN, of the 28 samples considered here, 9 were < $LoQ_{NH_4^+}$ (32%), 2 < $LoD_{NH_4^+}$ (7%) and one < $LoD_{NO_3^-}$ (4%). All four samples for which TN was below LoQ were also < $LoQ_{NH_4^+}$.

Total organic nitrogen was calculated as $[TON] = [TN] - [NO_3^-] - [NH_4^+]$ (all factors in mg$_N$ L^{-1}), while the fraction of organic nitrogen (in %) was obtained as $f_{TON} = [TON]/[TN] \cdot 100$.

Results

Table S3 and Figure S6 summarize the results of our analyses. As an addition to the main text, we highlight below a few details on TOC and nitrogen analyses.

	**Average**	**Median**	**Range**	**R^2 (x vs. cond)[a]**	**R^2 (x vs. $\Sigma\alpha_{200-400}$)**
[TOC] (mg$_C$ L^{-1})	12 ± 14	8.8[b]	1.6 – 74[b]	0.14 (0.40)[c]	0.10 (0.55)[c]
Conductivity (µS cm^{-1})	15 ± 7	15	1 – 27	–	0.84
$\Sigma\alpha_{200-400}$ (cm^{-1})	20 ± 10	22	3.2 – 38	0.84	–
pH	5.7 ± 0.5	5.7	4.9 – 6.8	0.02	0.07
[TN] (mg$_N$ L^{-1})	1.6 ± 0.7	1.6	0.34 – 3.1	0.81	0.79
[TON] (mg$_N$ L^{-1})	1.1 ± 0.5	1.1	0.25 – 2.1	0.85	0.74
[NH_4^+] (mg$_N$ L^{-1})	0.34 ± 0.22	0.33	0.04 – 0.85	0.27	0.40
[NO_3^-] (mg$_N$ L^{-1})	0.15 ± 0.10	0.15	0 – 0.41	0.60	0.60
f_{TON} (%)	70 ± 10	68	51 – 93	0.001	0.05

Table S3 Overview of quantitative parameters for the 20-needle soak samples collected from the ponderosa pine in the Arboretum from May 22 to June 27, 2023. [a] Associated plots in Figure S6 (grey circles). [b] Equivalent to 3.3 µg cm^{-2} (median) and 0.6 – 28 µg cm^{-2} (range). [c] Values in parenthesis are obtained by excluding samples with [TOC] > 24 mg$_C$ L^{-1} (N = 3); see text.

TOC outliers. TOC is positively correlated with conductivity and $\Sigma\alpha_{200-400}$ but some datapoints fall clearly out of the average trend (Figure S6A). Among these outliers, we identified most soaks collected in the CSU Main Campus outside the Arboretum (yellow datapoints), the single outlier collected in the Horsetooth Mountain Open Space (light brown datapoints), and the first replicate of the "Solvent-to-leaf contact time" test (grey circle). These samples have higher than average TOC values and were tentatively identified as being less aromatic and the corresponding trees being less healthy than average based on other data (see main text).

Leaf surface coverage for dissolved organics. TOC data (in mg$_C$ L^{-1}) in Table S3 were converted to surface coverage (Γ, in µg cm^{-2}) via eq S3.

$$\Gamma = \frac{[\mathrm{TOC}]}{f_{\mathrm{TOC/TM}}} \cdot \frac{V_{\mathrm{soak}}}{A_{\mathrm{soak}}} \cdot 10^3, \qquad \text{S3}$$

where $f_{\mathrm{TOC/TM}}$ ≈ 0.5 mg_C mg^{-1} (typical value for aquatic dissolved organic matter),[17] V_{soak} = 10 mL = 0.01 L, A_{soak} = 53 cm^2 (value for 20 needles), and 10^3 is a conversion factor from mg to µg. This calculation yields Γ = 0.6 – 28 µg cm^{-2} (range), with a median value of 3.3 µg cm^{-2}, while the highest-measured TOC value of 150 mg_C L^{-1} (Figure 6, main text) converts to a surface coverage of 57 µg cm^{-2}.

Total and organic nitrogen. Although we detected nitrogen in all soaks and composite raindrops, we did not include this data in the main text due to close-to-detection limit TN concentrations. In future work, we recommend using *undiluted* samples for nitrogen analyses.

Despite these limitations, we observed positive correlations for TN vs. conductivity (R^2 = 0.81; Figure S6C) and TON vs. conductivity (R^2 = 0.85; Figure S6D), while the behavior of inorganic nitrogen species was less predictable, especially for NH_4^+ (Figure S6E-F). This result suggests that organic compounds drive the overall behavior of needle soak nitrogen – as further hinted by the strong relationship between TN and TON (R^2 = 0.92; insert in Figure S6D).

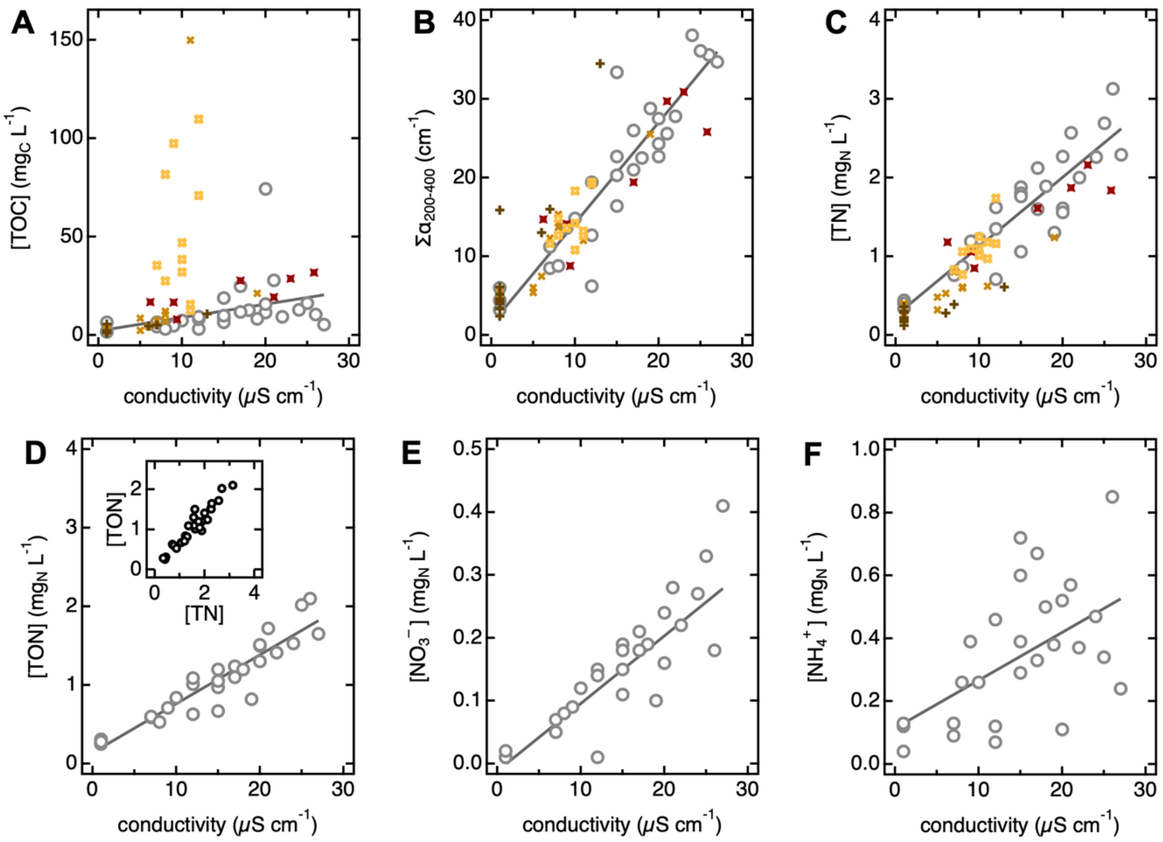

Figure S6 Trends in TOC (**A**), $\Sigma\alpha_{200-400}$ (**B**), TN (**C**), TON (**D**), nitrate (**E**), and ammonium (**F**) as a function of conductivity for 20-needle soaks collected from the ponderosa pine in the Arboretum (grey circles; $N = 28$). The dark grey line is the linear regression whose R^2 is reported in Table S3. Similar plots were obtained using $\Sigma\alpha_{200-400}$ as the x-axis (R^2 in Table S3, plots not shown). The colored dots in panels **A** – **C** are data from other tests reported for comparison. Specifically, red points are averages from the "Solvent-to-leaf contact time" test (20 needles, various soaking times), while brown and yellow symbols are from the "Location and plant-to-plant variability" test (20 needles for 5 min, various plants; dark brown: L1; light brown: L2; yellow: L3). Information about these other tests is in Texts S7 and S8. The insert in panel **D** shows the correlation between TON and TN ($R^2 = 0.92$; slope = 0.66; $N = 28$).

Text S7: Details on tests evaluating sampling variables

General note on conductivity. Average conductivity values include 0s and 1s μS cm^{-1} data, which we measured for samples having low average conductivity (< 5 μS cm^{-1}). As a reference, MilliQ water (blank) was *always* 0 or 1 μS cm^{-1}, so any average value > 1 μS cm^{-1} should be considered higher than the blank.

General note on $SUVA_{254}$. Samples with TOC < LoQ showed considerably higher $SUVA_{254}$ values than replicates collected under the same conditions but with TOC > LoQ and were not included in average calculations of $SUVA_{254}$ values.

Solvent: Solvent volume-to-leaf area ratio

Data source We evaluated this variable with samples from other tests. For the 5-needles datapoint, we used the two replicates collected on June 6 for the "On-plant vs. detached" needle test (on-plant only); for the 10-needle datapoint, we employed the three replicates collected on June 8 to test the effect of stomata closure (only open stomata); for the 20-needle datapoint, we utilized the two replicates collected on June 6 for the "Solvent-to-leaf contact time" test (5 min soaks only) and the first sample of the "Meteorological conditions" test (June 8). All samples were collected on June 6 or 8 from the ponderosa pine in the Arboretum by soaking *N* living needles in 10 mL of MilliQ water for 5 min in a pre-leached falcon tube.

Results We observed a linear correlation between number of needles and all extensive variables (i.e., TOC, conductivity, and $\Sigma\alpha_{200-400}$), while the quality of the organic material as assessed by absorption spectrum parameters was overall constant (Figure S7). $SUVA_{254}$ was the only absorption spectrum parameter showing a significant increase (R^2 = 0.994; Figure S7E) yet remaining within the average range of (1.0 ± 0.5) L mg_C^{-1} m^{-1}. This test is described in detail in the main text.

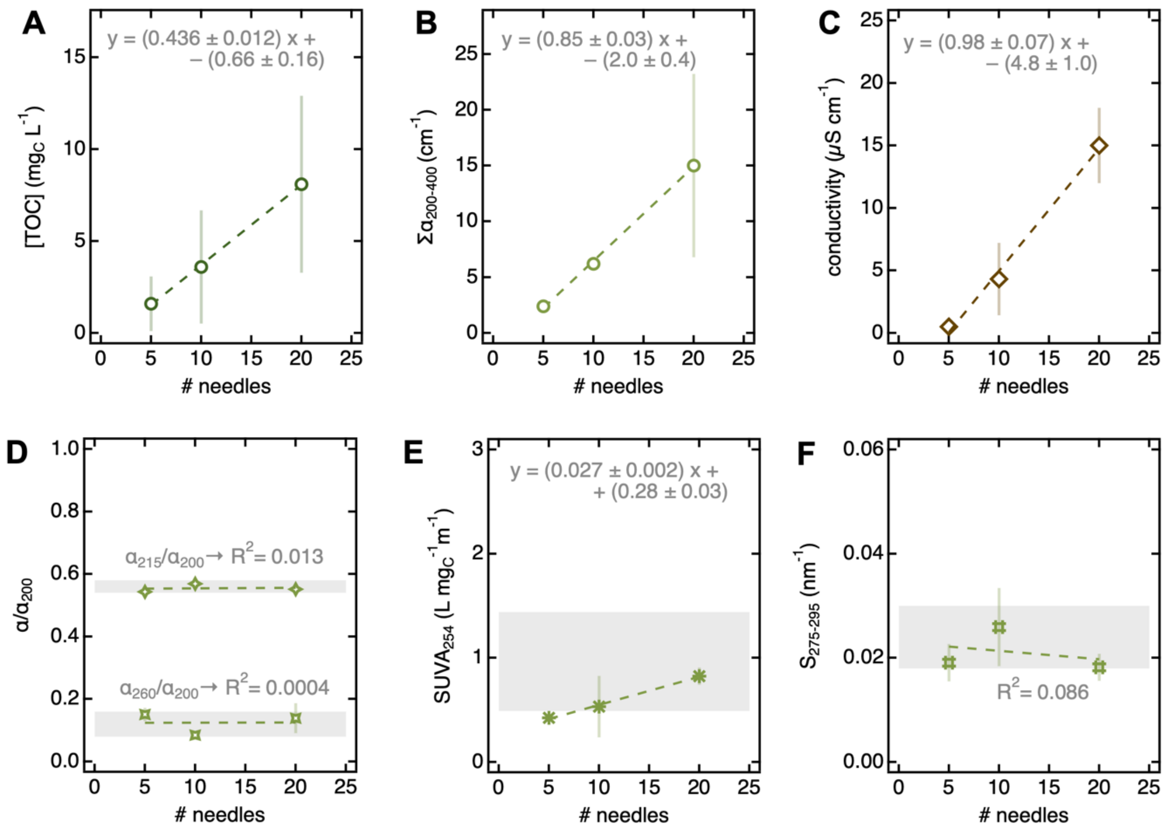

Figure S7 Changes in TOC (**A**), $\Sigma\alpha_{200-400}$ (**B**), conductivity (**C**), absorption coefficient ratios (**D**), $SUVA_{254}$ (**E**), and spectral slope (**F**) as a function of needle number. Each datapoint is the average of two or three independent samples collected on June 6 or 8 and error bars are standard deviations (within the symbol when not visible). $SUVA_{254}$, calculations include only datapoints with TOC > LoQ – thus, the 5-needle is an individual datapoint (no error bar), while the other two are averages of two instead of three values. Dashed lines are linear regressions; for significant correlations ($R^2 > 0.994$), we report the equation of the line rather than the R^2. pH values were measured only for 20-needle soaks and are not shown. Grey areas in panels **D-F** are typical ranges for ponderosa pine soaks (Table 1).

Solvent: Rain vs. MilliQ water

Experiment We selected 6 × 20-needle bundles on the ponderosa pine in the Arboretum. The first three bundles were soaked in 10 mL of MilliQ water for 5 min, while the remaining were immersed for in 10 mL of rainwater for 5 min. Rainwater was collected in Fort Collins on June 4 using a 500 mL Nalgene bottle secured in vertical position on the top of a metal bar. This sample was kept frozen in the collection bottle until use. All soak replicates were collected on June 21.

Results We detected minimal variations in the quantity of organic and inorganic material and no significant changes in organics' quality when using rain instead of MilliQ water as soak solvent (Table S4). Conductivity was the only parameter that varied significantly between treatments (p = 0.028) – however, this difference could be fully justified by differences in background solvent's conductivity.

Parameter	Different?	Needle soak		Background[a]	
		Rainwater	MilliQ (control)	Rainwater	MilliQ (control)
TOC (mg_C L^{-1})		13 ± 11	7.3 ± 0.8	1.7	< LoD
$\Sigma\alpha_{200-400}$ (cm^{-1})		23 ± 5	19 ± 4	4.0	< 0
Conductivity (μS cm^{-1})	* (0.028)	17.3 ± 0.6	12.3 ± 2.5	4.5	0.5
pH		6.1 ± 0.4	5.9 ± 0.3	n.m.	n.m.
$\alpha_{215}/\alpha_{200}$		0.552 ± 0.014	0.557 ± 0.008	0.55	n.c.
$\alpha_{260}/\alpha_{200}$		0.09 ± 0.03	0.094 ± 0.004	n.c.	n.c.
$SUVA_{254}$ (L mg_C^{-1} m^{-1})		0.81 ± 0.23	1.06 ± 0.29	n.c.	n.c.
$S_{275-295}$ (nm^{-1})		0.027 ± 0.007	0.028 ± 0.004	n.c.	n.c.

Table S4 Effect of solvent type on needle soak chemistry. The asterisk marks variables significantly different between treatment (p-value in parenthesis). All values are an average of three replicates collected on a single day, and the error is their standard deviation. Needle soak data were not corrected for background, which is reported in grey for comparison. [a] Based on analysis of a single sample, so standard deviations are not reported. Legend: n.m. = not measured; n.c. = not calculated (due to lack of absorption).

Sampling approach: Solvent-to-leaf contact time

Experiment For each treatment replicate, we prepared 7 × 20-needle bundles on the ponderosa pine in the Arboretum. The bundles were soaked in 10 mL of MilliQ for increasing times, namely 5 sec, 1 min, 2 min, 5 min, 15 min, 30 min, and 60 min. While soaking, we did not make any effort to screen the solution from light nor to limit temperature increase caused by light or heat. We collected a total of five treatment replicates: one on May 22, two on June 6, and two on June 26.

Results We observed a systematic increase in TOC, $\Sigma\alpha_{200-400}$, and conductivity as a function of soaking time (Figure 4 and S8A-C), while the quality of the organic material as assessed by absorption spectrum parameters and the solution pH were overall constant (Figures S8D-E and S9). This test is discussed in detail in the main text.

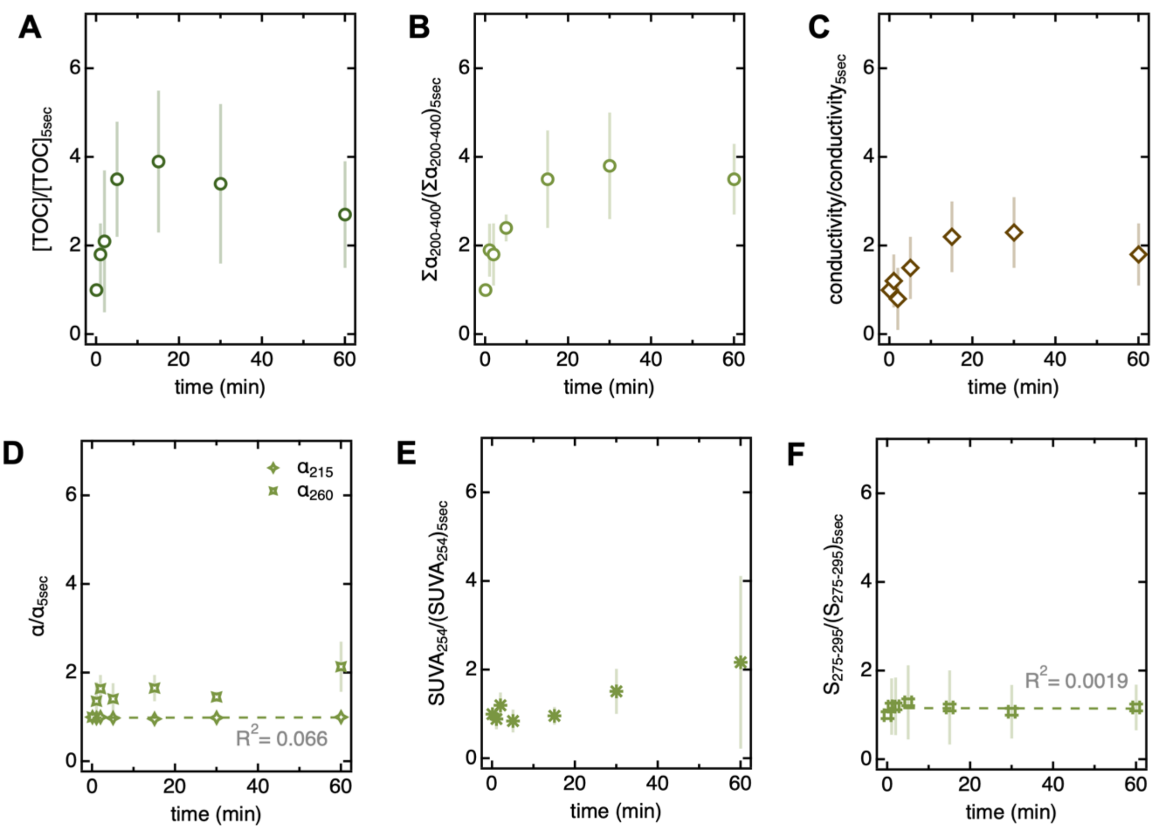

Figure S8 Daily normalized changes in TOC (**A**), $\Sigma\alpha_{200-400}$ (**B**), conductivity (**C**), absorption coefficient ratios (**D**), $SUVA_{254}$ (**E**), and spectral slope (**F**) as a function of soaking time (20 needles in 10 mL of MilliQ water). The reference used for normalization is the 5 sec datapoint. Error bars are standard deviations and are within the symbol when not visible (5 sec datapoints do not have error bars by definition). Average $SUVA_{254}$ values do not include points with TOC < LoQ – thus, each point is the average of ≥ three replicates. Dashed lines are linear regressions and are reported only for variables with unequivocal lack of variation as a function of time.

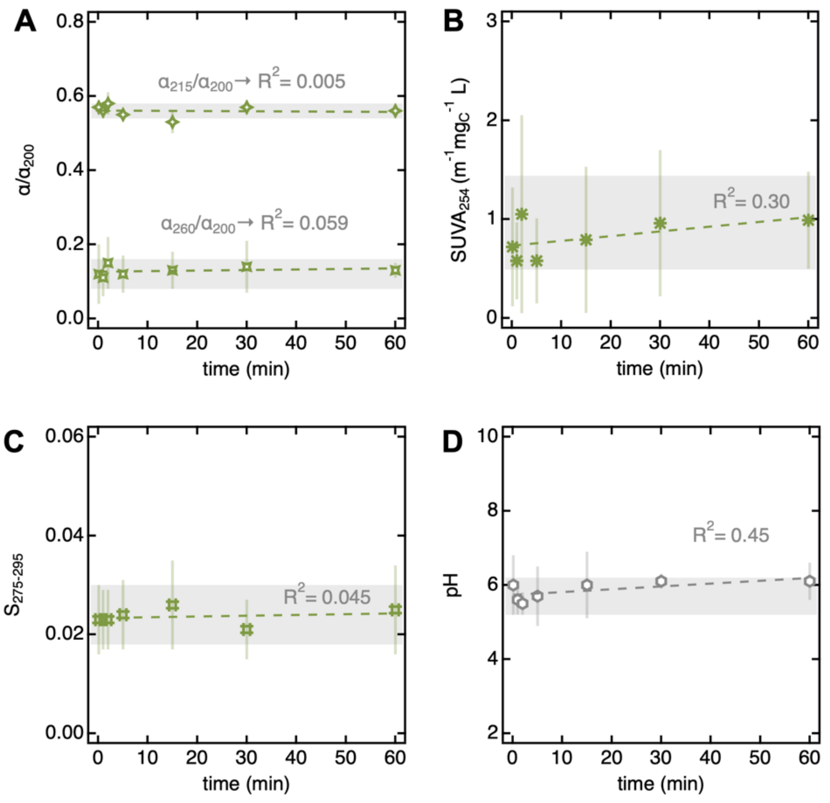

Figure S9 Changes in absorption coefficient ratios (**A**), $SUVA_{254}$ (**B**), spectral slope (**C**), and pH (**D**) as a function of soaking time (20 needles in 10 mL of MilliQ water). Each datapoint is the average of five sample replicates collected over three different days, and error bars are standard deviations. Average $SUVA_{254}$ do not include values with TOC < LoQ, thus each point is the average of ≥ three replicates. Dashed lines are linear regression lines. Typical ranges for ponderosa pine needle soaks are depicted as shaded grey areas (based on Table 1).

Sampling approach: Sonication

Experiment We collected living and dead needles from the ponderosa pine in the Arboretum (dead needles were taken from the branches, not the ground). We brought the needles to the lab, isolated them from the fascicles, removed the sheath, and wrapped the ending with a small piece of parafilm. We then submerged 5 living needles in 10 mL of MilliQ water for 5 min in a pre-leached tube, while a second group of 5 living needles was immersed in 10 mL of MilliQ water and sonicated for 5 min in a Branson 3510 ultrasonic cleaner. The same procedure was repeated for the dead needles. In total, we obtained five replicates for both living and dead needles over three days, i.e., one on May 22, two on June 6, and two on June 26. To evaluate the effect of sonication on the water-soluble material, we also prepared an additional 5-needle soak with living needles. 5

mL of this solution was sonicated for 5 min (*without needles*), while the remaining 5 mL were used as control.

Results Two lines of evidence indicate that sonication damages the needle, releasing additional material in solution. The first is that sonicated needles often displayed horizontal dark stripes that were never observed in non-sonicated samples (Figure S10). This feature was more evident in dead vs. living needles. The second is that a few absorption spectrum parameters changed in sonicated needles as compared to the controls. $\Sigma\alpha_{200-400}$ and $\alpha_{215}/\alpha_{200}$ were statistically higher in sonicated dead needles as compared to non-sonicated ones (p = 0.014 and 0.038, respectively), while $\alpha_{260}/\alpha_{200}$increased significantly in sonicated living needles (p = 0.024; Table S5). After normalizing for day-to-day differences (blue values in Table S5), $\Sigma\alpha_{200-400}$ and $\alpha_{260}/\alpha_{200}$ increased in all treatments; normalized $SUVA_{254}$ was also slightly higher in sonicated samples as compared to the controls, especially in living needles. Daily normalized TOC values were higher in sonicated dead needles as compared to the control, further hinting that dead cells break more easily than living ones. In one replicate of the sonicated living needle, we detected the two features associated to needle damage in this tree (at 245 and 275 nm), whereas two replicates of sonicated dead needles had a small but detectable feature at 275 nm. Collectively, this data hints to the release of organic material during sonication, part of which may be coming from inside the needle. Dead needles are more prone to this process than living ones.

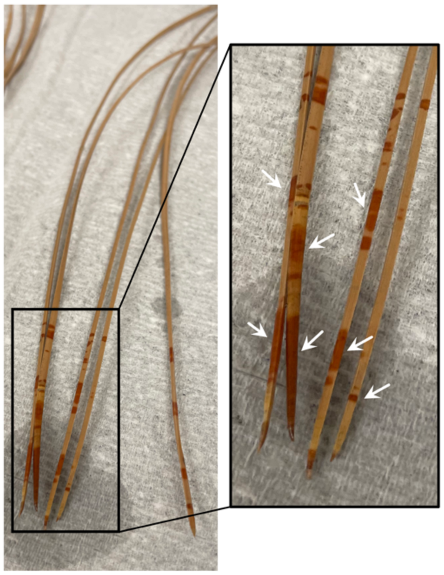

Figure S10 Dead needles after sonication. Darker stripes were always observed after sonication in dead needles (white arrows), while in living needles, they were more difficult to detect due to the minimal difference in color.

We tested the effect of sonication alone on the leached material (from living needles only), finding no difference in TOC and absorption parameters before and after treatment (conductivity was not measured). This fact supports our hypothesis that sonication helps release more organic material from the needle rather than modifying compounds leached from water alone. We note that this conclusion may change for longer

sonication times or higher sonication power, or for different plant species (which may have less robust cuticle or/and cell wall).

Parameter	Living			Dead		
	D?	Sonicated	Control	D?	Sonicated	Control
TOC (mg_C L^{-1})		7 ± 7<br>(1.7 ± 0.8)	4.2 ± 3.5	 †	15 ± 11<br>(3.1 ± 1.2)	6 ± 7
$\Sigma\alpha_{200-400}$ (cm^{-1})	 †	15 ± 15<br>(3.3 ± 2.2)	4.1 ± 1.8	*<br>†	39 ± 24<br>(7.0 ± 1.9)	5.3 ± 2.4
Conductivity (μS cm^{-1})		6 ± 7<br>(2.4 ± 2.6)	3.0 ± 3.2		7 ± 7<br>(1.4 ± 0.7)	4.6 ± 3.4
$\alpha_{215}/\alpha_{200}$		0.59 ± 0.06<br>(1.04 ± 0.11)	0.57 ± 0.04	*<br>†	0.60 ± 0.03<br>(1.06 ± 0.03)	0.56 ± 0.02
$\alpha_{260}/\alpha_{200}$	*<br>†	0.24 ± 0.07<br>(1.8 ± 0.7)	0.14 ± 0.04	 †	0.19 ± 0.07<br>(1.40 ± 0.21)	0.14 ± 0.06
$SUVA_{254}$ (L mg_C^{-1} m^{-1})	 †	1.1 ± 0.4<br>(2.4 ± 0.7)	0.49 ± 0.23		1.5 ± 0.6<br>(3.7 ± 3.1)	0.45 ± 0.27
$S_{275-295}$ (nm^{-1})		0.027±0.020<br>(0.8 ± 0.6)	0.034±0.010		0.018±0.007<br>(1.0 ± 0.6)	0.020±0.008

Table S5 Effect of sonication on the chemistry of soaks prepared with living (detached) and dead needles. As data were collected over three days, we also report daily normalized averages calculated using the non-sonicated control as reference (blue values). These normalized values are dimensionless. Variables that increased significantly after sonication are marked with a black asterisk (*, original values; *p*-values are in the text) or a blue symbol (†, normalized values) in columns marked as "D?" (meaning "Different?"). All values are an average of five replicates collected on three days, and the error is their standard deviation; $SUVA_{254}$ are averages of three replicates. pH was not measured because most conductivity values were < 5 μS cm^{-1}. Results on conductivity are uncertain due to the high prevalence of data < 5 μS cm^{-1} (50%).

Sampling approach: Filtration

Data source and further sample treatment This test relies on samples collected for other experiments. Data collected for the "Storage condition" test was employed to assess the impact of filtration on conductivity, pH, and absorption parameters (Text S5). In addition, we used some samples collected on May 22 to evaluate how filtration affects TOC, namely the first replicates of the "Solvent-to-leaf contact time", "On-plant vs. detached", and "Sonication" tests. An aliquot of each of these samples was filtered into an acid-cleaned TOC vial and diluted gravimetrically to 20 mL. Filtration was performed with 30 mm syringe filters as outlined in Text S5 ("Syringe filtration protocols to limit sample loss"). The associated unfiltered samples were analyzed with the same 10-fold dilution during the same instrument run and used as controls.

Results We observed minimal changes in the chemistry of filtered vs. unfiltered samples (Table S6). The lack of change in TOC between filtered and unfiltered samples was particularly surprising given the insoluble residues we often noticed at the bottom of collection tubes. We hypothesized these to be large particles that sediment rapidly and were inadvertently excluded from analyses of unfiltered samples.

Normalized parameter	Different?	Filtered	*N*
TOC		1.01 ± 0.06	8
$\Sigma\alpha_{200-400}$		1.03 ± 0.05	10
Conductivity		1.04 ± 0.04	8
pH		0.96 ± 0.05	8
$\alpha_{215}/\alpha_{200}$		0.99 ± 0.01	10
$\alpha_{260}/\alpha_{200}$		0.99 ± 0.05	10
$S_{275-295}$		1.03 ± 0.07	10

Table S6 Impact of filtration on soak chemistry. The table shows only averages of normalized parameters (i.e., $y/y_{filtered}$), so no p-values are reported. *N* is the number of samples used for the assessment. $SUVA_{254}$ could not be assessed.

Sampling approach: Freezing

Experiment Living needles from the ponderosa pine in the Arboretum were collected, brought to the lab, isolated from the fascicles, and their ending freed from the sheath and wrapped in a small piece of parafilm. 5 living needles were then submerged in 10 mL of MilliQ water for 5 min in a pre-leached falcon tube (control). A second group of 5 needles was wrapped in aluminum foil and stored in the freezer (– 20˚C) for 24 to 48 h. After this time, the needles were allowed to warm to room temperature and soaked in 10 mL of MilliQ water for 5 min. In total, we obtained five replicates of the two treatments over three different days (one on May 22, two on June 6, and two on June 26).

Results We observed no statistical difference in TOC, conductivity, and $\Sigma\alpha_{200-400}$ (Table S7) between treatments. Likewise, absorption spectra features were equal within their errors, indicating no evident changes in organic matter quality (Table S7). These conclusions may not be applicable to longer storage times, lower storage temperature, or different plant species.

Parameter	Different?	Frozen	Unfrozen (control)
TOC (mg_C L^{-1})		3.3 ± 3.7 (0.8 ± 0.4)	4.2 ± 3.5
$\Sigma\alpha_{200-400}$ (cm^{-1})		3.8 ± 1.3 (1.0 ± 0.3)	4.1 ± 1.8
Conductivity (μS cm^{-1})		2.4 ± 2.4 (1.3 ± 1.3)	3.0 ± 3.2
$\alpha_{215}/\alpha_{200}$		0.58 ± 0.03 (1.01 ± 0.05)	0.57 ± 0.04
$\alpha_{260}/\alpha_{200}$	(?)	0.11 ± 0.02 (0.8 ± 0.2)	0.14 ± 0.04
$SUVA_{254}$ (L mg_C^{-1} m^{-1})		0.72 ± 0.49 (1.6 ± 1.4)	0.49 ± 0.23
$S_{275\text{-}295}$ (nm^{-1})	(?)	0.021 ± 0.007 (0.7 ± 0.3)	0.034 ± 0.010

Table S7 Impact of needle freezing on soak chemistry. Blue values are corrected for day-to-day variability (reference: unfrozen sample); these values are dimensionless. All values are average of five replicates collected on three different days, and the error is their standard deviation. $SUVA_{254}$ is the average of three replicates only. pH was not measured because most replicates had conductivity < 5 μS cm^{-1}.

Leaf: Healthy vs. damaged

Experiment Living needles from the ponderosa pine in the Arboretum were brought to the lab (within 1 hour of collection), isolated from the fascicles, and their ending wrapped the ending with a small piece of parafilm. We prepared a control soak by immersing 5 healthy needles in 10 mL of MilliQ water for 5 min. We then took 5 other needles, broke and removed their tip, and immediately immersed them in 10 mL of MilliQ water for 5 min. In total, we obtained five replicates of the two treatments over three different days: one replicate on May 22, two on June 6, and two on June 26.

Results Breaking needles releases organic material in solution, as demonstrated by the significant increase in $\Sigma\alpha_{200-400}$ ($p < 0.0001$) and a substantial enhancement in normalized TOC in damaged vs. healthy needles (Table S8). We additionally noticed variations in organic matter *quality* as indicated by the significant decrease in $\alpha_{260}/\alpha_{200}$ ($p = 0.008$) and a moderate increase in normalized $\alpha_{215}/\alpha_{200}$. All damaged needle replicates consistently showed a small absorption feature at 275 nm, which was never observed in the corresponding controls. The *decrease* in $\alpha_{260}/\alpha_{200}$ associated with an *increase* in TOC, $\Sigma\alpha_{200-400}$, and, potentially, $\alpha_{215}/\alpha_{200}$ may underscore a release of aliphatic material absorbing < 250 nm. The large $SUVA_{254}$ errors of this

dataset do not allow one to draw confident conclusions on this hypothesis. Conductivity also appeared consistently higher in damaged needles (5 out of 5 values > 5 µS cm^{-1}) as compared to healthy ones (2 out of 5 values > 5 µS cm^{-1}), but the low values prevent conclusive statements.

**Parameter**	**Different?**	**Damaged**	**Healthy (control)**
TOC (mg_C L^{-1})	†	7.6 ± 1.9<br>(3.0 ± 2.0)	4.2 ± 3.5
$\Sigma\alpha_{200-400}$ (cm^{-1})	* (< 0.0001)<br>†	14.9 ± 2.2<br>(4.4 ± 2.3)	4.1 ± 1.8
Conductivity (µS cm^{-1})	(0.040)	6.8 ± 1.3<br>(3.6 ± 2.8)	3.0 ± 3.2
$\alpha_{215}/\alpha_{200}$	(†)	0.61 ± 0.02<br>(1.08 ± 0.07)	0.57 ± 0.04
$\alpha_{260}/\alpha_{200}$	* (0.008)<br>†	0.076 ± 0.008<br>(0.57 ± 0.20)	0.14 ± 0.04
$SUVA_{254}$ (L mg_C^{-1} m^{-1})		0.62 ± 0.16<br>(1.2 ± 0.4)	0.49 ± 0.23
$S_{275-295}$ (nm^{-1})		0.039 ± 0.015<br>(1.3 ± 0.8)	0.034 ± 0.010

Table S8 Impact of needle damage on soak chemistry. Blue values are corrected for day-to-day variability (reference: healthy needle control); these values are dimensionless. Variables that changed significantly are marked with a black asterisk (*, original values; *p*-values in parenthesis) or a blue symbol (†, normalized values). All data are an average of five replicates collected on three different days, and the error is their standard deviation. $SUVA_{254}$ is the average of three replicates only. pH was not measured because most replicates had conductivity < 5 µS cm^{-1}.

Leaf: Living vs. dead

Experiment We collected living and dead needles from the ponderosa pine in the Arboretum. (Dead needles were taken from the branches, not the ground.) We isolated the needles from the fascicles, removed the sheath, and wrapped the ending with a small piece of parafilm. We then submerged 5 living needles in 10 mL of MilliQ water for 5 min in a pre-leached falcon tube (control); the same procedure was repeated for 5 dead needles. In total, we obtained five replicates of the two treatments over three different days, namely one replicate on May, two replicates on June 6, and two more on June 26. Needle manipulation and soaking were carried out in the lab.

Results We observed no difference between dead and living needles, before and after correcting for day-to-day variability (Table S9), with the only exception of $S_{275-295}$ (p = 0.040). This result

may hint that dead needles release higher molecular weight material as compared to living ones. We note that these results may change depending on the dead needle "age".

Parameter	Different?	Dead	Living (control)
TOC ($mg_C\ L^{-1}$)		6.4 ± 7.2 (1.4 ± 0.5)	4.2 ± 3.5
$\Sigma\alpha_{200-400}$ (cm^{-1})		5.3 ± 1.4 (1.3 ± 0.6)	4.1 ± 1.8
Conductivity ($\mu S\ cm^{-1}$)		4.6 ± 3.4 (2.5 ± 3.0)	3.0 ± 3.2
$\alpha_{215}/\alpha_{200}$		0.56 ± 0.02 (0.99 ± 0.06)	0.57 ± 0.04
$\alpha_{260}/\alpha_{200}$		0.14 ± 0.06 (1.0 ± 0.4)	0.14 ± 0.04
$SUVA_{254}$ ($L\ mg_C^{-1}\ m^{-1}$)		0.45 ± 0.27 (1.0 ± 0.7)	0.49 ± 0.23
$S_{275-295}$ (nm^{-1})	* (0.040) †	0.020 ± 0.008 (0.6 ± 0.3)	0.034 ± 0.010

Table S9 Effect of using dead vs. living needles on soak chemistry. Blue values are corrected for day-to-day variability (reference: living needle control); these values are dimensionless. Variables that changed significantly are marked with a black asterisk (*, original values; *p*-values in parenthesis) or a blue symbol (†, normalized values). All values are average of five replicates collected on three different days, and the error is their standard deviation. $SUVA_{254}$ is the average of three replicates only. pH was not measured because most replicates had conductivity < 5 $\mu S\ cm^{-1}$.

Leaf: On-plant vs. detached

Experiment We prepared a needle soak sample (5 needles in 10 mL of water for 5 min) and collected a few living needles from the ponderosa pine in the Arboretum. These needles were brought back to the lab, isolated from the fascicles, freed from the sheath, and their ending was wrapped in a small piece of parafilm. 5 of these needles were then submerged in 10 mL of MilliQ water for 5 min in a pre-leached falcon tube. This sample was prepared within one hour of needle collection. In total, we obtained five replicates of the two treatments over three different days, namely one replicate on May 22, two on June 6, and two on June 26.

Results We detected no variation in quantity and quality of material released in solution between the two treatments, before and after correcting for day-to-day variability (Table S10).

Parameter	Different?	Detached	On-plant (control)
TOC (mg_C L^{-1})		4.2 ± 3.5<br>(1.1 ± 0.8)	5.0 ± 3.6
$\Sigma\alpha_{200-400}$ (cm^{-1})		4.1 ± 1.8<br>(1.2 ± 0.4)	3.6 ± 1.3
Conductivity (µS cm^{-1})		3.0 ± 3.2<br>(2.3 ± 2.5)	1.8 ± 2.4
$\alpha_{215}/\alpha_{200}$		0.57 ± 0.04<br>(1.01 ± 0.11)	0.56 ± 0.02
$\alpha_{260}/\alpha_{200}$		0.14 ± 0.04<br>(1.07 ± 0.10)	0.13 ± 0.03
$SUVA_{254}$ (L mg_C^{-1} m^{-1})		0.49 ± 0.23<br>(1.6 ± 0.7)	0.33 ± 0.09
$S_{275-295}$ (nm^{-1})		0.034 ± 0.010<br>(1.3 ± 0.5)	0.027 ± 0.008

Table S10 Variation in the chemistry of soaks prepared in the field with on-plant needles and those prepared in the lab with detached needles. Blue values are corrected for day-to-day variability (reference: on-plant control); these values are dimensionless. All values are average of five replicates collected on three different days, and the error is their standard deviation. $SUVA_{254}$ is the average of three replicates only. pH was not measured because most replicates had conductivity < 5 µS cm^{-1}.

Leaf: Open vs. close stomata

Experiment We prepared 6 × 10-needle bundles on the ponderosa pine in the Arboretum. Three bundles were wrapped in aluminum foil for > 5 hours to induce stomatal closure,[18,19] whereas the remaining ones were left unwrapped. After this time, we used a portable photosynthesis system (PPS) to quantify stomatal conductivity and CO_2 assimilation on each bundle; immediately after, the needles were soaked in 10 mL of MilliQ water for 5 min. For "dark" bundles, we minimized light exposure by wrapping the PPS leaf chamber and the sampling tubes with aluminum foil. This experiment was performed on June 8.

Results Whether stomata are closed or open has no impact on soak chemistry (Table S11), suggesting that the material released in solution is not leached through open stomata. We note that 50% of the conductivity data was < 5 µS cm^{-1}, justifying the large relative error in the average value.

**Parameter**	**Different?**	**Close stomata**	**Open stomata (control)**
CO_2 assimilation (μmol m^{-2} s^{-1})	* (0.0035)	– (0.008 ± 0.016)	0.53 ± 0.15
Stomatal conductance (nmol m^{-2} s^{-1})	* (0.0096)	– (1.1 ± 1.5)	8.4 ± 3.2
TOC (mg_C L^{-1})		3.6 ± 3.0	3.6 ± 3.1
$\Sigma\alpha_{200-400}$ (cm^{-1})		8.4 ± 5.4	6.2 ± 0.5
Conductivity (μS cm^{-1})		5.3 ± 7.5	4.3 ± 2.9
$\alpha_{215}/\alpha_{200}$		0.59 ± 0.02	0.569 ± 0.004
$\alpha_{260}/\alpha_{200}$		0.09 ± 0.03	0.084 ± 0.008
$SUVA_{254}$ (L mg_C^{-1} m^{-1})		0.69 ± 0.06	0.53 ± 0.30
$S_{275-295}$ (nm^{-1})		0.022 ± 0.005	0.026 ± 0.008

Table S11 Effect of stomata closure on soak chemistry. The asterisk indicates data that are statistically different between replicates; the associated *p*-value is in parenthesis. All values are the average of three replicates collected on a single day, and the error is their standard deviation. pH was not measured due to low conductivity.

Text S8: Details on tests evaluating environmental variables

Meteorological conditions

Experiment We collected a 20-needle soaks each day between June 8 and June 27 from the ponderosa pine in the Arboretum (20 needles soaked in 10 mL of MilliQ water for 5 min; no sample replicates were collected). The samples were kept frozen until analyses.

Additional data and error estimation Filled datapoints in Figures 5, S11, and S13 show the same type of sample (i.e., 20 needles in 10 mL of MilliQ water for 5 min) collected from the same tree but for other tests. For June 21, we used the data from the "Rain vs. MilliQ water" test (MilliQ water only; $N = 3$), while for June 26 we employed two replicates of the "Solvent-to-leaf contact time" test (5 min only; $N = 2$). For each day, we pooled all available data ($N \geq 3$), calculated average ($\bar{y}$) and standard deviation (σ_y) for each variable and used this information to compute relative errors ($\sigma_y/\bar{y} \cdot 100$, in %). Error bars are obtained using the *average* relative error over the two sampling days, which was 38% for TOC, 26% for $\Sigma\alpha_{200-400}$, and 25% for conductivity.

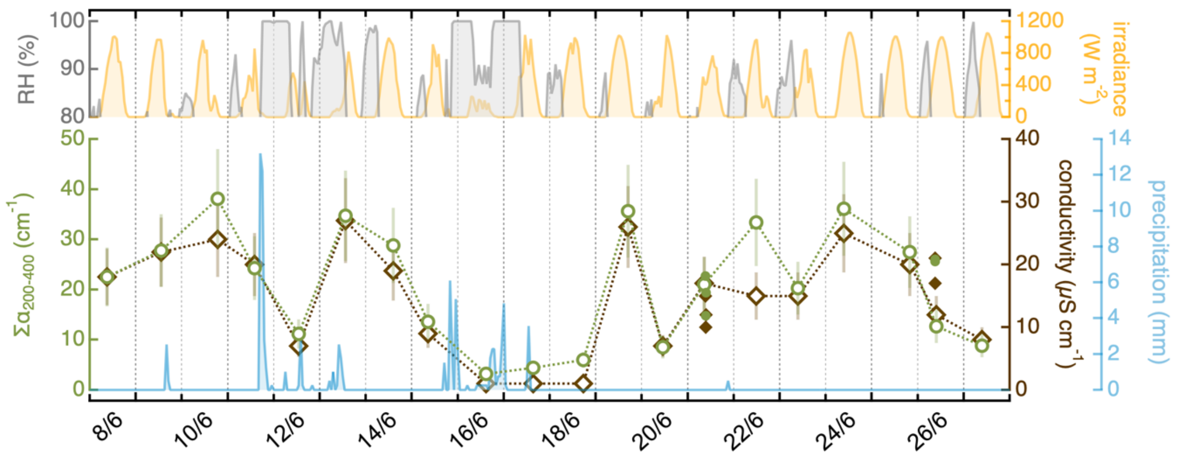

Figure S11 Correlation between meteorological conditions and $\Sigma\alpha_{200-400}$ (light green trace) for 20-needle soaks collected from the ponderosa pine in the Arboretum between June 8 and June 27 (complement of Figure 5). Conductivity, precipitation, irradiance, and RH are the same as Figure 5 and are shown for reference. Filled dots are 20-needle, 5-min soak samples collected on the same ponderosa pine for a different test. Data points are individual samples and error bars are estimated from the average relative errors in $\Sigma\alpha_{200-400}$ (26%).

Results This experiment is described in detail in the main text; supplementary data is in Figures S11 – S13.

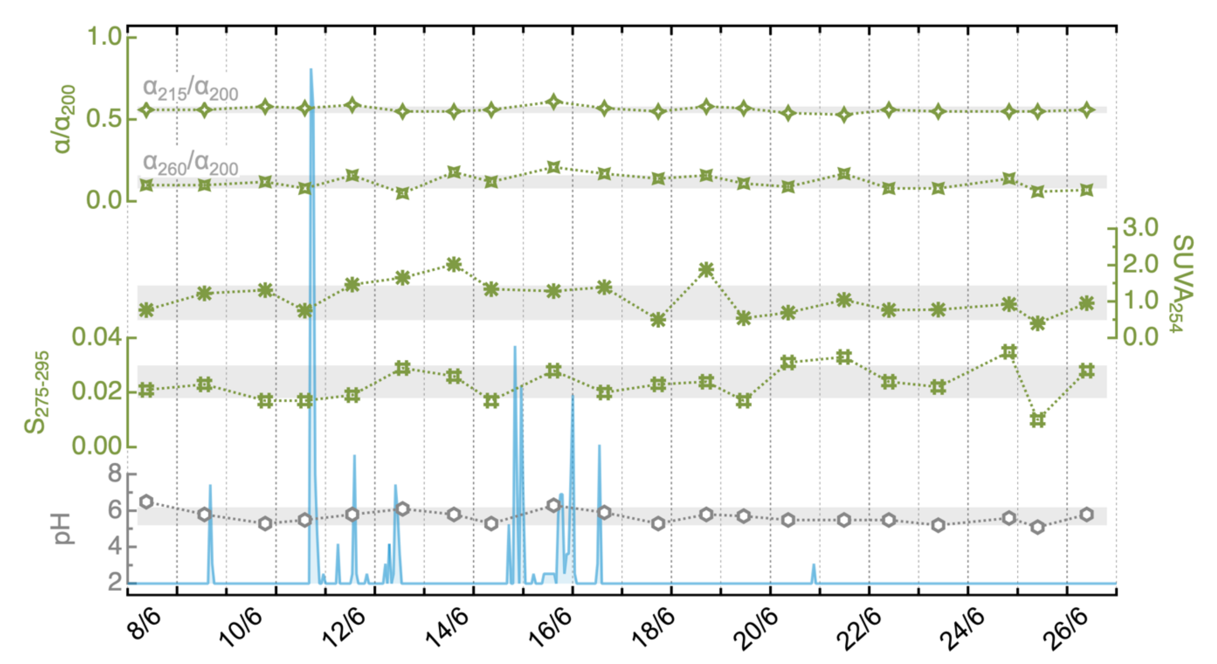

Figure S12 Trends in organic matter quality (light green) and pH (grey) as a function of sampling day. Each datapoint is an individual 20-needle soak collected from the ponderosa pine in the Arboretum. Units for $SUVA_{254}$ and $S_{275\text{-}295}$ are L mg_C^{-1} m^{-1} and nm^{-1}, respectively, and are omitted for clarity. The light blue trace is the cumulative precipitation during the sampling period (as in Figures 5 and S11) and is shown only for reference. Average values during the sampling periods are $\alpha_{215}/\alpha_{200} = 0.56 \pm 0.02$, $\alpha_{260}/\alpha_{200} = 0.12 \pm 0.04$, $SUVA_{254} = (1.06 \pm 0.47)$ L mg_C^{-1} m^{-1}, $S_{275\text{-}295} = (0.023 \pm 0.006)$ nm^{-1}, and pH = 5.6 ± 0.4 ($N = 20$). These values are statistically equivalent to typical data for ponderosa pine needle soaks ($p \gg 0.05$ for all variables; shaded grey areas, see Table 1 for reference).

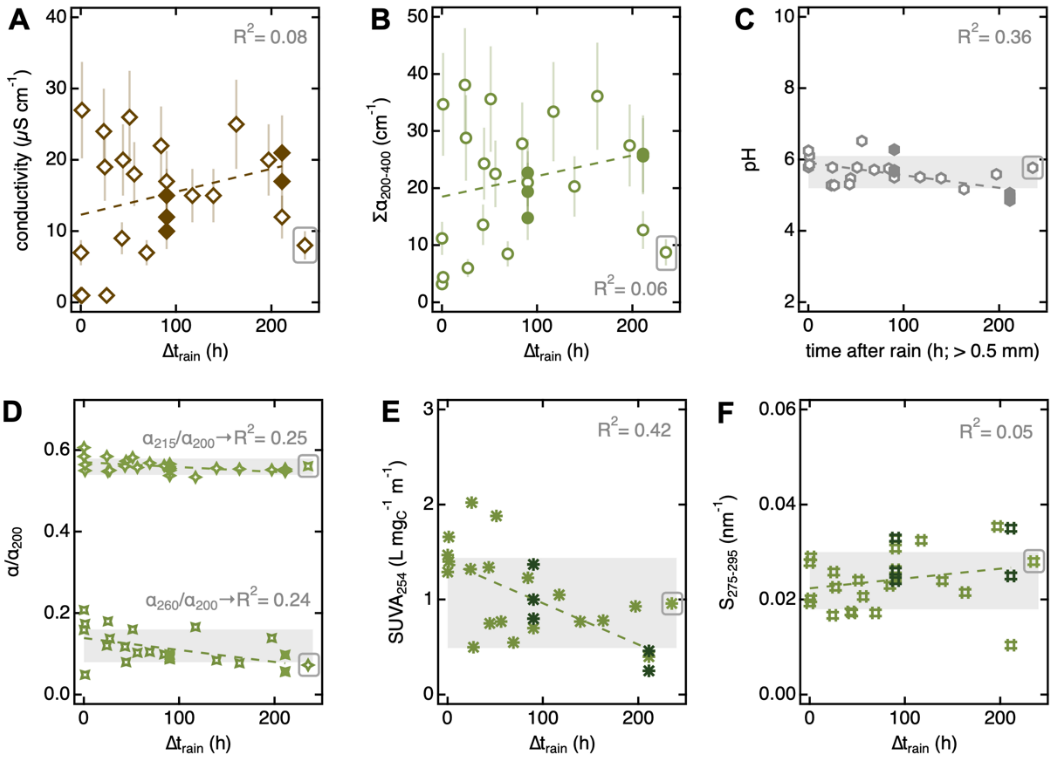

Figure S13 Trends in conductivity (**A**), $\Sigma\alpha_{200-400}$ (**B**), pH (**C**), absorption ratios (**D**), $SUVA_{254}$ (**E**), and $S_{275\text{-}295}$ (**F**) as a function of time from the last rain event (> 0.5 mm; Δt_{rain}). Datapoints are individual replicates and dashed lines are linear regressions. Error bars in panels **A** and **B** are estimated from average relative errors in conductivity (25%) and $\Sigma\alpha_{200-400}$ (26%), respectively, while the grey areas in panels **C** – **F** are typical ranges for ponderosa pine needle soaks (see Table 1). The fit considers only samples collected between June 8 and 26 (including those from other tests; filled or darker markers); the soak taken on June 27 is excluded from the fit (datapoint in the grey boxes).

Location and plant-to-plant variability

Experiment We collected 20-needle soaks in triplicates from four different ponderosa pines in three different locations (Table S1). Pines at the CSU Mountain Campus were sampled on June 18, while samples at the Horsetooth Mountain Open Space and CSU Main Campus (outside the Arboretum) were collected on June 19 (see also Text S1). In each location, we prepared an experimental blank (10 mL of MilliQ water in a pre-leached falcon tube) and measured the circumference at breast height for tree age estimation. When sampling in rural locations, we minimized the time samples stayed at ambient temperature by storing them in a chilled container during car trips (2.5 hours to reach the CSU Mountain Campus and 30 min for the Horsetooth

Open Space). Samples collected at the Horsetooth Open Space remained at ambient temperature for an additional 0.5 – 1.5 hours, the time needed to hike from the sampling location to our car. All samples were kept frozen until analyses.

Results This test is described in detail in the main text. Additional data is provided in Table S12 and Figure S14.

				**Different? (*p*-value)**	
**Parameter**	**L1**	**L2**	**L3**	**L1 vs. L3**	**L2 vs. L3**
TOC (mg_C L^{-1})	3.3 ± 2.8<br>(2.0 ± 1.0)	7.4 ± 5.7<br>(6.3 ± 3.7)	49 ± 33	< 0.0001<br>(0.0004)	0.0003<br>(0.0011)
$\Sigma\alpha_{200-400}$ (cm^{-1})	9.4 ± 9.3<br>(5.2 ± 3.1)	10.5 ± 6.5	15.1 ± 3.3	(< 0.0001)	0.040
Conductivity<br>(μS cm^{-1})	2.9 ± 3.8<br>(1.6 ± 1.7)	6.3 ± 5.1<br>(5.4 ± 2.8)	11.8 ± 6.9<br>(10.0 ± 1.7)	0.0007<br>(< 0.0001)	0.034<br>(*)
pH	5.5 ± 0.2	5.7 ± 0.2<br>(5.6 ± 0.2)	5.6 ± 0.4<br>(5.4 ± 0.2)		
$\alpha_{215}/\alpha_{200}$	0.57 ± 0.05	0.57 ± 0.02	0.55 ± 0.02		
$\alpha_{260}/\alpha_{200}$	0.19 ± 0.06	0.13 ± 0.03	0.11 ± 0.03<br>(0.10 ± 0.02)	0.0004<br>(0.0004)	(0.018)
$SUVA_{254}$<br>(L mg_C^{-1} m^{-1})	1.78 ± 0.26[a]	0.66 ± 0.27<br>(0.75 ± 0.14)	0.20 ± 0.17<br>(0.17 ± 0.10)	< 0.0001<br>(< 0.0001)	0.0003<br>(*)
$S_{275-295}$ (nm^{-1})	0.021 ± 0.004	0.020 ± 0.006<br>(0.018 ± 0.003)	0.012 ± 0.004[b]	< 0.0001	0.004<br>(0.0031)

Table S12 Average chemistry parameters for needle soaks collected at the CSU Mountain Campus (**L1**), Horsetooth Mountain Open Space (**L2**), and CSU Main Campus (**L3**). Values are averages of 12 sample replicates collected on four trees (three replicates per tree) and the error is their standard deviation. Grey values are averages excluding trees responsible for outliers (identified as shown in Figure 6; excluded samples are marked with asterisks in Figures 6 and S14). If not reported, no tree outliers were found for that specific parameter. The right columns report *p*-values of variables identified as statistically different ($p < 0.05$) in **L1** vs. **L3** and **L2** vs. **L3**. Values in grey and parenthesis are obtained excluding tree outliers. [a] To be considered indicative, as only half of the samples had TOC > LoQ and were included in the average (N = 6). [b] Four samples were excluded from the average due to a feature at 310 nm that interfered to spectral slope calculation (N = 8).

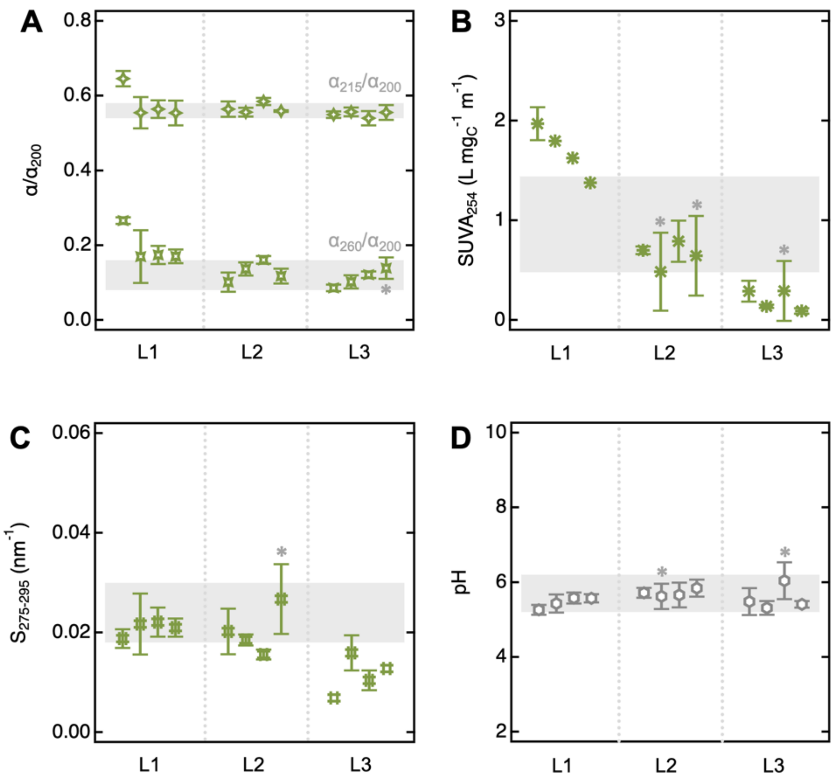

Figure S14 Organic matter quality proxies (**A** – **C**; light green) and pH (**D**, grey) for ponderosa pines sampled at the CSU Mountain Campus (**L1**), Horsetooth Mountain Open Space (**L2**), and CSU Main Campus (**L3**). Each datapoint is the average of three sample replicates (20 needles in 10 mL of MilliQ for 5 min) and the error bar is the standard deviation. In **L1**, we report only one $SUVA_{254}$ value for all plants (but the first) because TOC < LoD for the remaining sample replicates. Likewise, in **L3**, we report only one $S_{275-295}$ for the first and last plant (data without error bars) because the other two replicates have distinct features at 310 nm that interfered with spectral slope calculations. Grey shaded areas are typical ranges obtained for the ponderosa pine in the Arboretum (see Table 1). Data marked with a grey asterisk were responsible for at least one outlier (defined by descriptive statistic; see main text) and were excluded from average calculations (grey values in Table S12).

References

(1) *About the Fort Collins Weather Station*. https://climate.colostate.edu/~autowx/fclwx_about.php (accessed 2024-01-11).

(2) *CSU Mountain Campus Instrumentation*. https://datavis.warnercnr.colostate.edu/instrumentation/ (accessed 2024-01-11).

(3) *Fort Collins Weather Station Data Access*. https://climate.colostate.edu/~autowx/fclwx_access.php (accessed 2024-01-11).

(4) *CSU Mountain Campus Instrumentation*. https://datavis.warnercnr.colostate.edu/instrumentation/station_data.cfm (accessed 2024-01-11).

(5) *Pinus ponderosa Dougl*. https://www.srs.fs.usda.gov/pubs/misc/ag_654/volume_1/pinus/ponderosa.htm (accessed 2024-01-11).

(6) Stubbins, A.; Silva, L. M.; Dittmar, T.; Van Stan, J. T. Molecular and Optical Properties of Tree-Derived Dissolved Organic Matter in Throughfall and Stemflow from Live Oaks and Eastern Red Cedar. *Front. Earth Sci.* **2017**, *5*, 22. https://doi.org/10.3389/feart.2017.00022.

(7) Van Stan, J. T.; Stubbins, A. Tree-DOM: Dissolved Organic Matter in Throughfall and Stemflow. *Limnol. Oceanogr. Lett.* **2018**, *3* (3), 199–214. https://doi.org/10.1002/lol2.10059.

(8) Chen, H.; Tsai, K.-P.; Su, Q.; Chow, A. T.; Wang, J.-J. Throughfall Dissolved Organic Matter as a Terrestrial Disinfection Byproduct Precursor. *ACS Earth Space Chem.* **2019**, *3* (8), 1603–1613. https://doi.org/10.1021/acsearthspacechem.9b00088.

(9) Dibley, M.; Jaffe, A.; O'Brien, R. E. Molecular Insights into Dissolved Organic Matter in Natural Dew Water: Biogrime Films on Leaf Surfaces. *ACS Earth Space Chem.* **2022**, *6* (3), 775–787. https://doi.org/10.1021/acsearthspacechem.1c00445.

(10) Ossola, R.; Tolu, J.; Clerc, B.; Erickson, P. R.; Winkel, L. H. E.; McNeill, K. Photochemical Production of Sulfate and Methanesulfonic Acid from Dissolved Organic Sulfur. *Environ. Sci. Technol.* **2019**, *53* (22), 13191–13200. https://doi.org/10.1021/acs.est.9b04721.

(11) Minor, E. C.; Swenson, M. M.; Mattson, B. M.; Oyler, A. R. Structural Characterization of Dissolved Organic Matter: A Review of Current Techniques for Isolation and Analysis. *Environ. Sci. Process. Impacts* **2014**, *16* (9), 2064–2079. https://doi.org/10.1039/C4EM00062E.

(12) Li, P.; Hur, J. Utilization of UV-Vis Spectroscopy and Related Data Analyses for Dissolved Organic Matter (DOM) Studies: A Review. *Crit. Rev. Environ. Sci. Technol.* **2017**, *47* (3), 131–154. https://doi.org/10.1080/10643389.2017.1309186.

(13) Helms, J. R.; Stubbins, A.; Ritchie, J. D.; Minor, E. C.; Kieber, D. J.; Mopper, K. Absorption Spectral Slopes and Slope Ratios as Indicators of Molecular Weight, Source, and Photobleaching of Chromophoric Dissolved Organic Matter. *Limnol. Oceanogr.* **2008**, *53* (3), 955–969. https://doi.org/10.4319/lo.2008.53.3.0955.

(14) Weishaar, J. L.; Aiken, G. R.; Bergamaschi, B. A.; Fram, M. S.; Fujii, R.; Mopper, K. Evaluation of Specific Ultraviolet Absorbance as an Indicator of the Chemical Composition and Reactivity of Dissolved Organic Carbon. *Environ. Sci. Technol.* **2003**, *37* (20), 4702–4708. https://doi.org/10.1021/es030360x.

(15) D'Andrilli, J.; Cooper, W. T.; Foreman, C. M.; Marshall, A. G. An Ultrahigh-Resolution Mass Spectrometry Index to Estimate Natural Organic Matter Lability. *Rapid Commun. Mass Spectrom.* **2015**, *29* (24), 2385–2401. https://doi.org/10.1002/rcm.7400.

(16) Riches, M.; Lee, D.; Farmer, D. K. Simultaneous Leaf-Level Measurement of Trace Gas Emissions and Photosynthesis with a Portable Photosynthesis System. *Atmospheric Meas. Tech.* **2020**, *13* (8), 4123–4139. https://doi.org/10.5194/amt-13-4123-2020.

(17) *Elemental Compositions and Stable Isotopic Ratios of IHSS Samples | IHSS*. https://humic-substances.org/elemental-compositions-and-stable-isotopic-ratios-of-ihss-samples/ (accessed 2023-09-07).

(18) Daszkowska-Golec, A.; Szarejko, I. Open or Close the Gate – Stomata Action Under the Control of Phytohormones in Drought Stress Conditions. *Front. Plant Sci.* **2013**, *4*, 138. https://doi.org/10.3389/fpls.2013.00138.

(19) Murchie, E. H.; Lawson, T. Chlorophyll Fluorescence Analysis: A Guide to Good Practice and Understanding Some New Applications. *J. Exp. Bot.* **2013**, *64* (13), 3983–3998. https://doi.org/10.1093/jxb/ert208.
